# Genetic constraints in genes exhibiting splicing plasticity in facultative diapause

**DOI:** 10.1038/s41437-024-00669-2

**Published:** 2024-01-30

**Authors:** Rachel A. Steward, Peter Pruisscher, Kevin T. Roberts, Christopher W. Wheat

**Affiliations:** 1 Zoology Department, Stockholm University, Stockholm, Sweden; 2 Biology Department, Lund University, Lund, Sweden; 3 Science for Life Laboratory, Department of Microbiology, Tumor and Cell Biology, Karolinska Institute, Stockholm, Sweden

**Keywords:** Genetic variation, Evolutionary ecology, RNA splicing, Gene expression

## Abstract

Phenotypic plasticity is produced and maintained by processes regulating the transcriptome. While differential gene expression is among the most important of these processes, relatively little is known about other sources of transcriptional variation. Previous work suggests that alternative splicing plays an extensive and functionally unique role in transcriptional plasticity, though plastically spliced genes may be more constrained than the remainder of expressed genes. In this study, we explore the relationship between expression and splicing plasticity, along with the genetic diversity in those genes, in an ecologically consequential polyphenism: facultative diapause. Using 96 samples spread over two tissues and 10 timepoints, we compare the extent of differential splicing and expression between diapausing and direct developing pupae of the butterfly *Pieris napi*. Splicing differs strongly between diapausing and direct developing trajectories but alters a smaller and functionally unique set of genes compared to differential expression. We further test the hypothesis that among these expressed loci, plastically spliced genes are likely to experience the strongest purifying selection to maintain seasonally plastic phenotypes. Genes with unique transcriptional changes through diapause consistently had the lowest nucleotide diversity, and this effect was consistently stronger among genes that were differentially spliced compared to those with just differential expression through diapause. Further, the strength of negative selection was higher in the population expressing diapause every generation. Our results suggest that maintenance of the molecular mechanisms involved in diapause progression, including post-transcriptional modifications, are highly conserved and likely to experience genetic constraints, especially in northern populations of *P. napi*.

## Introduction

Phenotypic plasticity, wherein a single genotype can produce different phenotypes in different environmental conditions, is a ubiquitous response to environmental variability (West-Eberhard 2003; Ghalambor et al. 2007). Plastic phenotypes are produced through perception of environmental cues, transduction of these cues as internal signals and morphological, physiological or behavioral responses (Nijhout 2003; Lafuente and Beldade 2019; van der Burg and Reed 2021). Both the production and maintenance of plastic phenotypes rely on processes regulating protein expression (Schlichting and Smith 2002), and while increased phenotypic plasticity has been associated with greater variation in gene expression (Leung et al. 2020) numerous post-transcriptional dynamics also mediate protein expression levels (Liu et al. 2016). However, the role other sources of transcriptional, and ultimately translational, variation play in phenotypic plasticity remain poorly understood.

Growing evidence suggests that alternative splicing is an important axis of transcriptional variation contributing to phenotypic plasticity (Marden 2008; Verta and Jacobs 2022; Wright et al. 2022). Large transcriptome-wide splicing differences between plastic phenotypes have recently been uncovered in a wide array of non-model species (Aamodt 2008; Grantham and Brisson 2018; Healy and Schulte 2019; Martin Anduaga et al. 2019; Lang et al. 2020; Thorstensen et al. 2021; Steward et al. 2022; Tian and Monteiro 2022). Differential splicing between plastic phenotypes is an important complement to whole-gene expression variation because it increases transcriptional complexity, control, and flexibility. Although fewer genes tend to be differentially spliced than differentially expressed between plastic phenotypes, splicing often affects a unique set of genes with nonoverlapping functions (Grantham and Brisson 2018; Steward et al. 2022).

Adaptive phenotypic plasticity is more likely to evolve when fluctuations in the environment are predictable and inductive cues are tightly correlated with future environments (Ezard et al. 2014; Leung et al. 2020). Although plasticity can buffer the genome from selection (Buckley and Kingsolver 2021), populations in these predictable, fluctuating environments experience similarly strong selection as in constant environments (Rescan et al. 2021). Strong selection for plasticity with reliable cues predictive of future conditions should lead to the fixation and maintenance of variants in regulatory cascades that are critical for translating environmental cues into optimal phenotypes. This prediction is difficult to test for gene expression plasticity because these regulatory loci are generally unknown. Depending on the genetic architecture of the phenotype, selection acting upon expression plasticity may target the *cis-*regulatory elements of genes, which often lie well up or downstream from genes whose expression they regulate (Cramer 2019; Mantica and Irimia 2022). Furthermore, genes with environmentally sensitive expression represent a small proportion of differentially expressed genes between alternative plastic phenotypes. In contrast, the majority of differentially expressed genes in the production and maintenance of these phenotypes arises through *trans-*regulation of gene regulatory networks. As a result, variation in a single locus can alter numerous downstream genes, especially at later developmental stages, making it difficult predict whether any single gene is likely to be the target of selection upon phenotypic plasticity.

In contrast to differentially expressed genes, genes exhibiting plasticity in alternative splicing may be particularly likely targets of selection acting upon expression plasticity because the *cis-*regulatory mechanisms necessary for splice-site recognition lie within the gene itself. Although alternative splicing occurs at splice sites on the boundaries of exons, context-specific splicing is largely determined by silencers and enhancers located within flanking exons and introns (Goren et al. 2006; Wang et al. 2006; Ule and Blencowe 2019; Mantica and Irimia 2022). Thus, strong selection to maintain phenotypic plasticity should lead to purifying selection at these regulatory loci, with direct consequences for genetic diversity within the gene body itself. Indeed, such effects have been seen in genes exhibiting alternative splicing differences between sexes in birds (Rogers et al. 2021), where autosomal genes routinely find themselves in alternative phenotypic states (e.g. male or female). Genes exhibiting splicing differences between alternating seasonal phenotypes in butterflies also exhibited decreased genetic diversity, with the greatest decrease among splicing events expected to be under the greatest *cis*-regulatory control (Steward et al. 2022). We aim to test whether populations that vary in their degree of phenotypic plasticity also harbor different levels of genetic diversity in genes with differential splicing between alternative phenotypes.

Here, we explore facultative diapause, a form of plasticity that helps organisms respond to seasonal variation. Diapause is a pre-programmed state of arrested development, during which organisms remain largely unresponsive to external stimuli (Koštál 2006). Organisms that exhibit facultative diapause can either induce diapause or continue with normal development, depending on environmental cues (Koštál 2006; Wilsterman et al. 2021). For many insects, this is a critical adaptation to temperate environments as it enables individuals to withstand long periods with limited resources and environmental stress, while also allowing rapid population growth when environmental conditions are advantageous (Wilsterman et al. 2021). Although the specifics of facultative diapause responses differ widely among insect taxa in life stage, phylogenetic background and environmental context, diapause is generally characterized by distinct physiological and transcriptomic changes that transition the individual through induction, maintenance and termination phases (Ragland et al. 2019; Dowle et al. 2020; Pruisscher et al. 2022). Variation in seasonality over space (e.g., changes in photoperiod along a latitudinal gradient) has resulted in local adaptation of diapause timing, with the consequence that populations often vary in voltinism, or the number of generations that can occur every year (Posledovich et al. 2015; Lindestad et al. 2019, 2020). Together, this suggests genes and regulatory networks underlying diapause are likely to experience different selection pressures in populations where facultative diapause is critical to survival compared to those undergoing a single developmental trajectory, with potential consequences for the underlying genetic diversity.

Insect diapause is characterized by drastic changes in gene expression, both in total and relative abundance (Poupardin et al. 2015; Kang et al. 2016; Ragland and Keep 2017; Koštál et al. 2017; Pruisscher et al. 2022). However, the role and extent of alternative splicing plasticity during diapause remains underexplored. Some evidence from single genes suggests that splice variants play important roles in diapause in various insect systems, including transcription factors (Chen et al. 2019; Abrieux et al. 2020), classical circadian clock genes (Barberà et al. 2017; Martin Anduaga et al. 2019; Abrieux et al. 2020), and diapause-specific neuropeptides (Zhang et al. 2014). Furthermore, splicing regulators, such as P-element somatic inhibitor (PSI) in *Drosophila melanogaster* and protein phosphatase 2A subunit A (PP2A-A) in *Helicoverpa armigera* are differentially expressed through dormancy and diapause, respectively (Tian and Xu 2013; Foley et al. 2019). Thus, insects are likely to exhibit changes in splicing profiles through diapause that differ compared to their direct developing counterparts. Despite the potential of alternative splicing as a mediator of diapause progression, genome wide analyses of splicing during diapause are noticeably lacking.

The butterfly *Pieris napi* has been a major focus of research into the physiology and evolution of diapause (Kivelä et al. 2015; Posledovich et al. 2015; Lehmann et al. 2017; Pruisscher et al. 2021; Nielsen et al. 2022; Süess et al. 2022). It is widespread through Eurasia and multivoltine in most of its range. *P. napi* overwinter as pupae, although diapause is induced by conditions experienced by late-stage larvae (Kivelä et al. 2015). At far northern latitudes, natural populations of *P. napi* have only one generation per year (Kivelä et al. 2015; Pruisscher et al. 2017), a consequence of the short summer seasons during which conditions allow mating, egg-laying and larval growth. Although facultative diapause exists in low frequencies in these populations, and direct development can be induced in lab conditions, these northern populations are functionally univoltine. This variation in diapause incidence presents an opportunity to test the hypothesis that differences in selection on plasticity can constrain the nucleotide diversity in genes that are differentially spliced among plastic phenotypes.

Previous analyses of transcript expression through pupal development found major changes across the phases of diapause (Pruisscher et al. 2022), but to what extent these patterns may be caused by alternative splicing remains unknown. Here, we revisit this rich transcriptomic dataset (96 samples over two tissues and 10 timepoints) to explore patterns of splicing plasticity, measured as exon expression and event expression, through diapause and direct development. We assess whether the patterns and extent of differential splicing differ from differentially expressed genes, representing a separate axis of transcriptional variation during diapause. Furthermore, we use pooled whole genome sequencing data to test our hypothesis that genes involved in splicing plasticity – in this case, genes that show different splicing patterns in diapause and direct development – experience greater genetic constraints compared to other genes exhibiting alternative splicing or whole gene expression differences. Finally, we compare genetic diversity and signatures of selection between populations with and without diapause plasticity in the wild to test whether positive or negative (i.e., purifying) selection acting upon plasticity impacts the genetic diversity of differentially spliced genes. Specifically, we predicted that nucleotide diversity in differentially spliced genes would be lower due to negative selection in a multivoltine population that exhibits both diapausing and direct developing phenotypes each generation. Instead, we found that both genes that were differentially spliced during diapause as well as those that were differentially expressed during diapause were constrained, but the degree of this constraint was stronger among spliced genes and this effect increased at northern latitudes. Thus, we found stronger negative selection associated with the diapause phenotype in the univoltine population, where diapause is critical to survival and under selection every generation.

## Methods

### RNAseq samples and experimental design

**Fig. 1:**
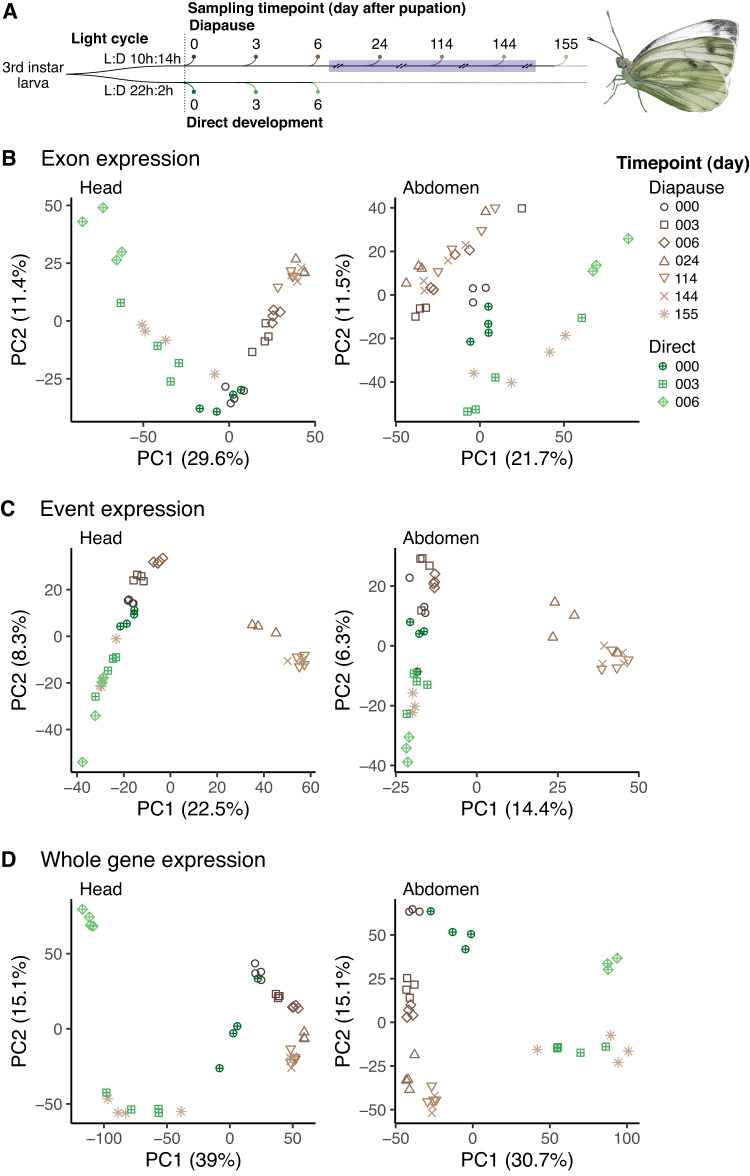
Transcriptomic changes through the development of diapausing and directly developing pupae. **A** Sampling time points (days after pupation) from the two developmental trajectories. Diapause or direct development was induced by exposing larvae to different photoperiods (Light:Dark cycles). To bring pupae out of diapause, diapausing pupae were exposed to cold temperatures between day 10 and 151 (blue rectangle) and kept in constant darkness between day 17 and 144. Diapausing larvae diverge from direct developing larvae at multiple levels of transcriptional variation: **B** exon expression, **C** event expression, and **D** whole gene expression.

We used RNAseq reads generated by Pruisscher et al. (2022), which are archived on NCBI under Bioproject PRJNA684967. Samples were taken from head and abdomen tissues of female pupae reared in conditions resulting in direct development (Light:Dark 22 h:2 h, 20 °C) or diapause (L:D 10 h:14 h, 20 °C; Fig. 1A). Direct developing individuals were sampled day 0, 3 and 6 of pupation. These butterflies spent about 10 days as pupae. Pupae in diapause were sampled at day 0, 3, 6, 24, 114, 144, and 155 after pupation. Diapausing *P. napi* require cold exposure to terminate diapause (Lehmann et al. 2016, 2017). Thus, these pupae were moved to 10 °C on day 10 after pupation, and 2 °C after day 17. After sampling on day 144, temperatures were increased to 10 °C, and to 20 °C on day 151. Pupae sampled at day 155 experienced a total of four days at 20 C after the termination of endogenous diapause (2–4 months after pupation, Lehmann et al. 2016). Samples were flash frozen in liquid nitrogen between 10:00–13:00 to reduce of circadian variation in gene expression and post-transcriptional modifications. RNA was extracted, libraries constructed, and sequenced reads cleaned using standard protocols (Supplementary Methods; Pruisscher et al. 2022).

### RNAseq read mapping and quantification

We used a *Pieris napi* genome assembly from the Darwin Tree of Life Project (GCA_905231885.1; ilPieNapi1; Lohse et al. 2021). We produced an inhouse annotation for the ilPieNapi1 genome using the BRAKER2 pipeline (v.2.1.5, Lomsadze et al. 2005; Stanke et al. 2006, 2008; Ter-Hovhannisyan et al. 2008; Buchfink et al. 2015; Hoff et al. 2016, 2019; Brůna et al. 2021). We ran BRAKER2 in protein mode, using Arthropoda OrthoDB (v.10) reference proteins. We compared this genome annotation with two inhouse annotations and two accessed from Darwin Tree of Life but found that the BRAKER2 protein-based assembly was the most complete, with the fewest fragmented BUSCOs, a small proportion of single exon genes, and more total estimated transcripts (Supplementary methods; Supplementary Tables S1, S2). We decided to move forward with the protein-based annotation but acknowledge that the low number of estimated transcripts relative to genes may mean that we may miss some exon- and junction-level variation.

We mapped cleaned and trimmed mRNA libraries to the ilPieNapi1 genome using hisat2 (v.2.2.1, Kim et al. 2019). We specified reverse-strandedness, but otherwise used default mapping parameters (Supplementary Table S3). After sorting and indexing with Samtools (v.1.9; Li et al. 2009; Danecek et al. 2021), we counted reads mapping to exons in the *P. napi* protein-informed annotation using featureCounts (RSubread v. 3.16; Liao et al. 2014, 2019) with the meta-feature defined as the geneID. To quantify exon expression, we specified read counting at the exon feature level allowing for reads to overlap multiple features but excluding reads mapping to multiple locations or that did not map in proper pairs. To quantify whole gene expression, we specified read counting at the meta-feature level and did not allow reads to overlap multiple gene features. This produced counts for 123,638 exons and 16,449 genes. Ultimately, two samples were excluded in downstream analyses due to divergent expression of genes involved in spermatogenesis, suggesting they were incorrectly sexed during sampling.

#### Differential splicing analyses

We used two proxies for differential splicing: exon expression and event expression. Exon-based analyses detect departures of exon expression from within-gene averages, whereas event-based analyses detect evidence for specific types of alternative splicing from reads mapping across splice junctions. Thus, while the first metric can provide a gene-level estimate of splicing, the second allows for a more detailed view of splicing within genes. Furthermore, these metrics have slightly different biases at low sample sizes (Mehmood et al. 2020). Most exon-based approaches tend to have low false positive rates, but only detect a small fraction of differential exon expression. Event-based approaches often detect a greater proportion of differentially spliced genes at low sample sizes, but with a higher false discovery rate across all sample sizes.

##### Differential exon expression

We used edgeR (v.3.36.0; Lun et al. 2016; Chen et al. 2016) to compare exon expression between days and developmental conditions separately for the pupal head and abdomen samples. We filtered out exons that did not meet our read count criteria using the edgeR function *filterByExpr* and default parameter settings, leaving 96,740 exons (12188 genes) in the head and 91,096 exons (11,888 genes) in the abdomen. We calculated normalization factors (Trimmed Mean of M-values (TMM)-method), estimated dispersion and fit a quasi-likelihood negative binomial generalized log-linear model based on a design comparing all days in each developmental condition (diapause and direct developing). We ran a series of pairwise comparisons of exon expression using two-sided quasibinomial F-tests, using the edgeR function *diffSpliceDGE* with the Simes adjustment to identify genes with significantly different (alpha = 0.01, Benjamini-Hochberg adjustment for multiple comparisons) exon usage. To minimize multiple comparisons, we focused on a subset of pairwise tests that would characterize how expression changed through diapause and direct development. Specifically, we compared diapause 000 with direct 000 to identify differences in splicing at the start of pupation for both developmental trajectories. We then used day 0 as a reference to assess changes within each developmental trajectory, comparing diapause 000 with each of the remaining diapause time points (003, 006, 024, 114, 144, 155) and direct 000 with each of the remaining direct time points (003 and 006). We also compared every stepwise change in expression (e.g., 003 vs 006, 006 vs 024, etc.) within each developmental trajectory, for a total of 15 comparisons.

We explored patterns of exon expression using principal component analysis (PCA) in head and abdomen samples from diapausing and direct developing pupae. To capture variation in exon expression that accounted for overall gene expression, we first normalized exon counts using the natural log of the counts per million (CPM) using TMM normalization factors. A similar method was used to normalize exon counts for differential exon expression analysis, described above. We then calculated a “residual” within-gene expression value for each exon by calculating the average exon expression for each gene then subtracting this from the normalized expression from each exon. Positive “residual” expression values occur when an exon’s expression was higher than the within-gene average. A matrix of these values was analyzed using the *prcomp* function in the R package stats (v.4.0.1, R Core Team 2021).

##### Differential event expression

We used rMATS-turbo (Shen et al. 2014) to compare event expression for the same 15 comparisons described above. We began by running all mapped read files through the rMATS “prep” and “post” steps. This generated a single file of inclusion counts for all samples and all events, ensuring the same set of junctions was considered for all analyses. We then ran pairwise comparisons using rMATS “stat” mode, with the expression level difference cut-off (-cstat) set to 0.001. The output for each comparison was imported into R, where we used a custom script to filter out events found in fewer than three individuals in each group with at least five reads supporting the inclusion or exclusion form of each splice event. This was done for each comparison separately. Using this subset of events, we identified significantly differentially expressed events as those with an adjusted *p*-value below 0.01 and deltaPSI (difference in “percent spliced in”, or difference in percentage of reads supporting the exclusion form of the event between the two groups) of greater than 0.05. Genes were considered to have different event expression in each comparison if they contained at least one significant splicing event.

As with exon expression, we used PCA to characterize patterns of event expression across all conditions and time points for each tissue. Here, we used inclusion level calculated by rMATS in the prep and post steps. We focused on the 5000 events with the highest variance and without missing data. As diapause timepoints 024, 114 and 144 consistently had the lowest overall mRNA expression (Pruisscher et al. 2022), the PCAs likely fail to capture variation in genes not expressed at these time points.

### Differential gene expression

For pupal head and abdomen samples, whole gene counts were normalized and fit with a quasi-likelihood negative binomial generalized log-linear model as described for differential exon expression. We ran the same 15 pairwise comparisons of whole gene expression using two-sided quasibinomial F-test. Genes were considered significantly differentially expressed between treatments if they had an adjusted *p*-value (BH-method) less than 0.01. We also performed PCAs of the whole gene counts, normalized as described above.

### Further analyses of transcriptional variation

For all three measures of transcriptional variation (exon expression, event expression and whole gene expression) we used fuzzy c-means clustering, implemented with the R package MFuzz (Kumar and Futschik 2007), to cluster differentially expressed exons, events and whole genes. Clustering was performed on standardized (MFuzz::*standardise()*) exon counts, inclusion levels, and whole gene counts for individuals (rather than the average within group) and the number of clusters was selected using the minimum centroid distance estimated by the function *Dmin*. Optimum cluster size was determined by the inflection point in the minimum centroid distance in a sequence of 2–20. To explore potential functional differences among clusters, we performed gene set enrichment analyses (GSEA) using the R package topGO (Alexa and Rahnenfuhrer 2022) on each of the exon, event and gene expression clusters identified in the head and abdomen. GSEA tested for overrepresentation of GO terms using one-sided Fisher’s exact tests (parent-child algorithm). A threshold of *p* < 0.05 was set to identify significant GO terms describing biological processes and molecular functions.

### Poolseq read mapping and variant calling

Butterflies were collected from field sites near Kullaberg and Luleå, Sweden, in 2015. The Kullaberg pool comprised exclusively females (*n* = 24), while the Luleå pool comprised both males and females in unknown proportions (*n* = 30). DNA was extracted, libraries prepared and sequenced reads cleaned using standard protocols (Supplementary Methods). Trimmed reads were mapped to the reference assembly with BWA-mem (v.0.7.17; Li 2013) using default parameters. We converted .sam files to .bam files with Samtools (v.1.10, Li et al. 2009; Danecek et al. 2021), filtering for paired reads. We created a pileup file for the two populations, specifying minimum phred and mapq scores of 20. Following the PoPoolation (Kofler et al. 2011) pipeline, the pileup file was filtered for insertions and deletions, with a 5 bp window around indels, before it was converted to a sync file (Population2 v1201; Kofler et al. 2011) containing 227,315,635 sites. We also created pileups for each of the pools individually, filtered for indels, and further subsampled the pileups without replacement to a target coverage of 20 reads for sites with a minimum coverage of 4 reads and maximum coverage of 100 reads (PoPoolation, subsample-pileup.pl). Cutoffs were chosen based on the read depth distributions of filtered pileup files. These steps resulted in 160,336,333 sites in the Luleå pool and 193,611,537 sites in the Kullaberg pool.

### Population genomic analyses

The sync file was imported into R using the package poolfstat (Gautier et al. 2022), subsampled to retain variants within the 10–90^th^ read depth percentiles, corresponding roughly with the 20–100 read coverage thresholds implemented above. This filtering left 7,883,347 variants, which were used to calculate *F*_*ST*_ between the two populations in 100 SNP sliding windows. Because the Luleå pool was an unknown mix of male and female butterflies and because sex chromosomes have lower effective population sizes than autosomes and are therefore more susceptible to drift, we removed all genes on the Z chromosome from downstream analyses.

We used PoPoolation to calculate nucleotide diversity (π), divergence from neutrality (Tajima’s D), and the ratio of nonsynonymous to synonymous polymorphism (π_N_/π_S_) within gene bodies. Both π and Tajima’s D were calculated from the subsampled pileup files using the PoPoolation basic pipeline script variance-at-position.pl. To do this, we used the pre-mRNA sequence of the longest isoform for each gene in the annotation, extracted from the protein-based BRAKER2 annotation using AGAT (agat_sp_keep_longest_isoform.pl). π_N_/π_S_ was calculated for the CDS extracted from these mRNAs using PoPoolation basic pipeline script syn-nonsyn- position.pl. As above, we removed all genes on the Z chromosome from downstream analyses. We also removed single exon genes, both to make the differentially spliced and differentially expressed gene sets more comparable, and to reduce the risk of including transposable elements which are sometimes erroneously annotated as single exon coding genes. Ultimately, we compared π, Tajima’s D, and π_N_/π_S_ among 11,425 and 9,114 genes in the Luleå and Kullaberg pools, respectively.

### Population genomics and transcriptional variation

We grouped genes based on whether features (e.g., exons, events, genes) were differentially expressed through diapause, direct development, or both diapause and direct development. The remaining genes were classified as having no differential exon expression (“None”). We compared π, Tajima’s D, and π_N_/π_S_ between the genes in each of these groups using nonparametric Kruskal–Wallis tests, with effect size estimated using η^2^. Multiple comparisons between groups were made using Dunn’s tests with a BH correction. To account for associations between gene length, recombination rate and potential for differential splicing, we used the matchRanges function of the nullRanges package in R (Davis et al. 2023, Mu et al. 2023). We subsampled the set of non-differentially spliced genes to match the lengths, relative chromosomal position, and total sample sizes of differentially spliced gene sets and tested for differences between the groups as described above. Relative chromosomal position was used as a proxy for recombination rate, which is positively correlated with e.g., π. This is an appropriate proxy because previous work has shown that relative recombination rate follows a negative second-degree polynomial distribution across most chromosomes in *Pieris napi* (Neethiraj 2019). The matched subsampling process was repeated 1000 times and the results from the full gene sets were compared against those from the matched sets using the means of the KW-statistic, effect size, and Dunn’s multiple comparison statistics and *p*-values. Because the combined size of the differentially expressed gene sets exceeded that of the non-DE genes, we randomly subsampled 50% of each of the Diap., Both, and Dir. genes to create focal sets against which non-DE genes were matched. Statistical analyses were performed in R with the package rstatix (v.0.7.0; Kassambara 2023). Data wrangling and visualization were supported by the tidyverse (v.1.3.2; Wickham et al. 2019) and ggpubr (v.0.4.0; Kassambara 2023) packages.

## Results

### Splicing and expression patterns through pupal development

Transcriptional variation among diapausing and direct developing pupae was analyzed separately for head and abdomen samples using principal component analysis (PCA), providing an overview of the consistency and magnitude of biological variation. To assess parallels among axes of transcriptional variation, two different measures of differential splicing (exon expression and event expression) were contrasted with whole gene expression patterns. Considering both two approaches provides a deeper understanding of potential splicing variation.

Both measures of splicing captured similar patterns. For exon expression, the first two PC axes (cumulatively 41.0% and 33.2% of total variance in the head and abdomen, respectively) show strong divergence in expression between diapausing and direct developing samples starting on day 3 of diapause (Fig. 1B). This divergence increased in head samples through diapause day 144, but on day 155 exon expression abruptly diverged from the remaining diapause samples, clustering instead with direct development day 3. A similar pattern was observed in abdomen samples, with the exception that diapause days 3 to 144 all clustered together, rather than separating by day. Similarly, event expression PCAs clearly separated the two developmental trajectories, with the first two PC axes capturing similar amounts of the variation (cumulatively 30.8% in the head; 20.7% in the abdomen) (Fig. 1C). However, in both tissues PC1 strongly separated diapause days 24–144 from the remainder of the samples, while clustering all the other timepoints, suggesting a very strong, divergent signature of cold diapause from directly development.

In general terms, splicing pattens were surprisingly similar to whole gene expression results, as originally reported by Pruisscher et al. (2022) and which we replicated here for comparative purposes (Fig. 1D). Compared to splicing, however, the first two PC axes for whole-gene expression described more of the total variance (cumulatively 54.1% in the head; 45.8% in the abdomen). Samples clustered much more tightly by day within each developmental trajectory, such as days 3, 6, and 24 in the head. This suggests more distinct differences in gene expression than alternative splicing, both among days and between diapause and direct development. However, these PCAs may obscure more detailed patterns of transcriptional variation among genes, highlighting a need for the more detailed comparisons below.

### Differentially spliced genes in diapause and direct development

**Fig. 2:**
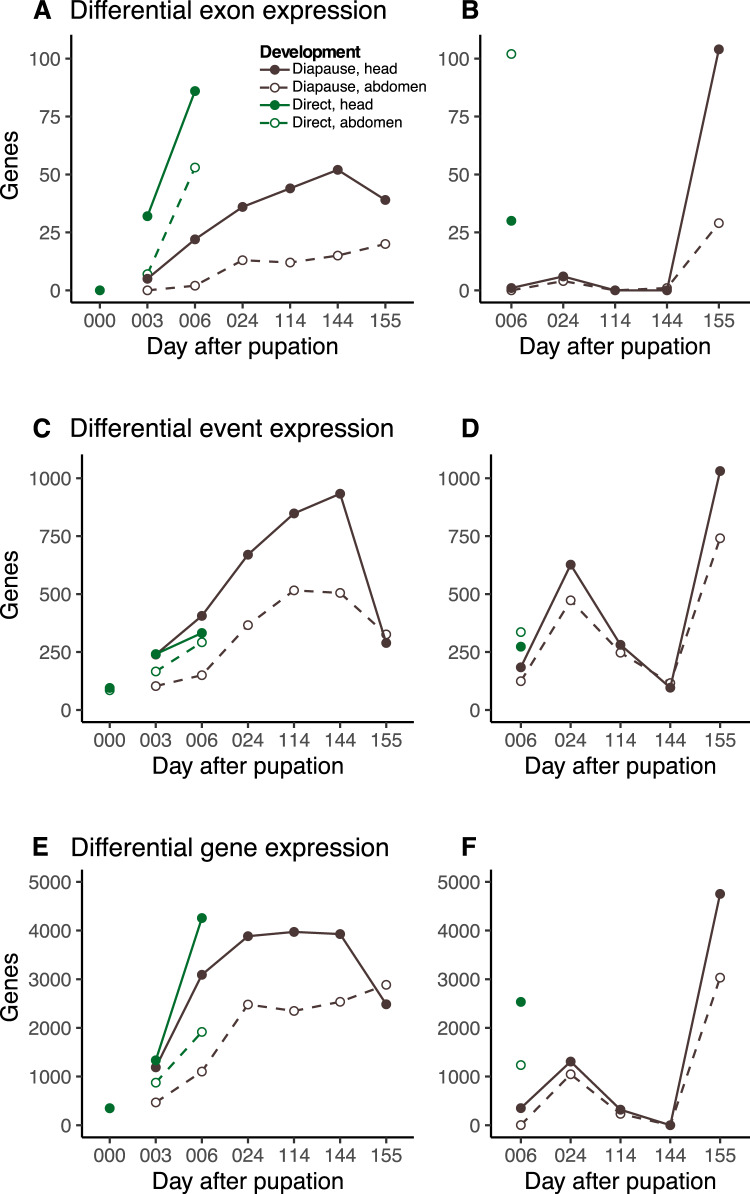
Total differentially spliced and differentially expressed genes across diapause and direct development. Reference analysis of differential (**A**) exon, (**C**) event and (**E**) gene expression through diapause and direct development, with the number of significant genes at each timepoint compared to day 0. Note that the results at day 0 (000) represent the number of genes differentially spliced or expressed between direct and diapause pupae on day 0. The remaining points connected by lines represent the comparison of indicated day (e.g., 003) vs. day 0. Stepwise analysis of differential (**B**) exon, (**D**) event and (**F**) gene expression through diapause and direct development, with the number of significant genes compared between adjacent timepoints, and labeled by the later developmental date (e.g. 006 shows results from 003 vs. 006).

For all three forms of transcriptomic variation (exon, event, and whole-gene expression), we found that there were very few differences between diapausing and direct developing samples on the day of pupation (day 000; Fig. 2A). To capture how expression changed through pupal development, we used two complimentary analyses of the data. First, we used day 000 as a reference to compare against subsequent time points within each developmental trajectory (e.g., 000 vs. 003, 000 vs. 006, 000 vs. 024, etc.; Fig. 2A). We also quantified transcriptional differences stepwise between subsequent time points, within the diapause and direct development trajectories (e.g., 003 vs. 006, 006 vs. 024, etc.; Fig. 2B). Together, these reference and stepwise approaches showed splicing differences slowly accumulated through diapause, increasing the magnitude of fold changes of exons and the change in percent-spliced-in (dPSI) of events through day 144. However, at day 155, there was a substantial shift in expression differences, seen in the large drop in differentially spliced or expressed genes at day 155 (Fig. 2A), as well as the spike in the direct comparison in differentially spliced or expressed genes between day 144 and 155 (Fig. 2B). Importantly, the altered expression observed at day 155 affected many of the same exons, events, and genes that were changing expression across the preceding timepoints, with day 155 of diapause exhibiting a general return to similar expression patterns observed on days 003 and 006 of direct development (Supplementary Fig. S1).

Relatively few genes were found to be differentially spliced through direct development using our event expression approach, posing the question of which types of splicing events might be changing over these timepoints and developmental pathways. We briefly investigated the specific types of splicing events detected through these developmental trajectories. We found that skipped exons (SE) and mutually exclusive exons (MXE) are most likely to be differentially expressed, regardless of which timepoints or trajectories were compared, and were overrepresented among the various splice types (Supplementary Table S4). Notably, these events are also the most likely to produce alternative functional isoforms (i.e. alternative protein products; Grantham and Brisson 2018; Steward et al. 2022; Wright et al. 2022).

Compared to either estimate of splicing, five times as many genes were found to have differential whole gene expression at each of the fifteen contrasts that were tested (Fig. 2). There was generally low overlap of differentially spliced and differentially expressed genes, with an average 24.9% (±5.6% 95% confidence interval) of genes containing differentially expressed events also being differentially expressed at the whole-gene level (Supplementary Table S5).

### Clustering of transcriptomic variation through diapause

We used fuzzy clustering to better illustrate the patterns of exon expression, resulting in eight and nine clusters in the head (ex.H1-8) and abdomen (ex.A1-9; Supplementary Table S6; Supplementary Fig. S2). Several clusters appear to be uniquely associated with the diapause phenotype. For example, cluster H3 contains exons that are downregulated at time points 3–144 in diapause in genes that are enriched for GO terms involved in reproduction and metabolism (e.g., oocyte localization, regulation of protein metabolic processes, establishment or maintenance of cell polarity; Supplementary Tables S6–8). In contrast, exons exclusively downregulated in direct development and day 155 of diapause (cluster ex.A7) were enriched for the molting cell cycle process and immune system processes (Supplementary Fig. S2B).

Overall, although patterns were similar among all three forms of transcriptomic variation, an order of magnitude more differentially spliced genes were detected using event expression than exon expression (Fig. 2; Supplementary Table S5). These events clustered into seven clusters in the head (ev.H1-6) and six in the abdomen (ev.A1-7; Supplementary Fig. S3). In both tissues, two of the clusters clearly identified events that were up and down regulated in response to the induction of the diapause cold treatment (ev.H1, ev.H7, ev.A3, ev.A6; Supplementary Fig. S3;). Cluster ev.H5 was especially interesting because it appears to be associated with splicing changes exclusive to the start of diapause (days 003-006), which were in genes highly enriched for mRNA processing, cytoskeletal protein binding and photoreceptor activity (Supplementary Fig. S3A).

### Genetic constraints on differentially spliced genes

**Fig. 3:**
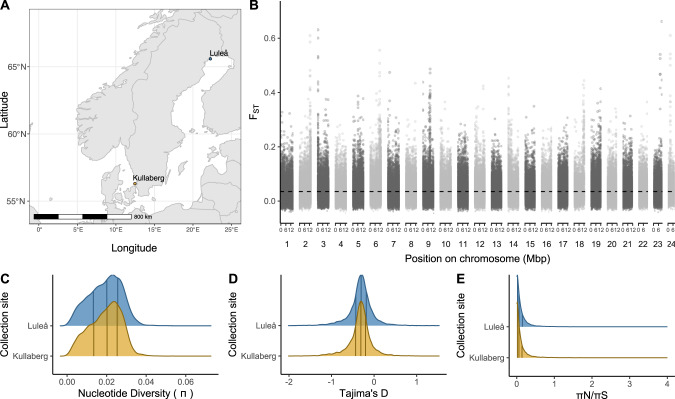
*Pieris napi* populations used to test genetic constraints. **A** Butterflies were collected from Kullaberg (yellow) and Luleå (blue) in Sweden. **B** Genome-wide genetic differentiation (Fst) between populations. Black and gray alternation delineates chromosomes. Distribution of (**C**) nucleotide diversity (π), (**D**) Tajima’s D and (**E**) πN/πS within populations. Vertical lines represent 25^th^, 50^th^ and 75^th^ quantiles. Genes mapping to the Z chromosome were excluded due to unknown composition of sexes in the Luleå pool.

We expected that selection on phenotypic plasticity should lead to strong purifying selection at relevant loci. We therefore predicted that genes exhibiting splicing unique to diapause or direct development would have lower genetic diversity and lower pN/pS levels, especially from populations that exhibit annual seasonal plasticity compared to those that exhibit only a single phenotype in the wild. To test this prediction, we compared the levels of genetic diversity across genes from a population of *P. napi* butterflies that produces both direct developing and diapausing individuals (a multivoltine population near Kullaberg, Sweden), with butterflies from a population without any direct generations (a univoltine population near Luleå, Sweden; Fig. 3A). While butterflies from this univoltine population have the capacity to develop directly, natural photoperiodic and temperature cues cause most larvae to develop into diapausing pupae (Posledovich et al. 2015).

Using pooled whole genome resequencing data, we estimated low genome-wide divergence between the two populations (mean Fst ± 95% CI = ~0.055 ± 0.002; Fig. 3B), contrasted with several large peaks of divergence across several chromosomes. Despite these differences, genome-wide distributions of nucleotide diversity (π),an assessment of the site frequency spectrum (SFS) using Tajima’s D, and the ratio of nonsynonymous to synonymous polymorphism within coding regions (π_N_/π_S_) were remarkably similar between the two populations, using estimates per individual gene locus (longest annotated isoform; Fig. 3C, D).

**Fig. 4:**
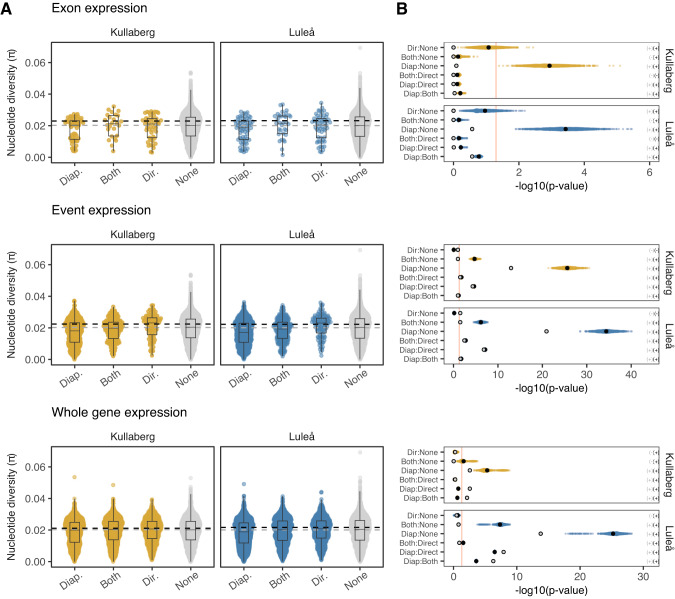
Nucleotide diversity (π) of genes with differential exon expression, event expression, and gene expression in the head. **A** π was compared among genes that were differentially spliced or expressed in diapausing timepoints only (Diap.), both diapausing and direct time points (Diap. & Dir), among direct timepoints only (Dir.) and in genes without transcriptional variation (None). Horizontal dashed lines represent median π for all ‘None’ genes (gray) and for matched gene sets (black). Matching the distribution of gene lengths, location along the chromosome (recombination rate proxy), and sample size with DS sets consistently increased the median nucleotide diversity in genes that were not DS (None). **B** Groups were compared with Kruskal–Wallis tests (Supplementary Table S9) with Dunn’s post-hoc pairwise comparisons corrected for multiple tests within each set (6 tests; Supplementary Table S10). Gray points show *p*-values (-log_10_ transformed) of Dunn’s multiple comparisons among full data, while yellow and blue points show *p*-values when comparing matched gene sets (*n* = 1000) for each population. Black points summarize means of these comparisons. The direction of the effect is shown in parentheses (gray = full data, black = matched ranges). Red lines represent significance thresholds of 0.05.

Using this population genomic data, we compared π between genes exhibiting significantly different splicing and expression patterns between diapause and direct development, for both populations. Specifically, we grouped genes with differential exon expression in diapause, in direct development, or both developmental trajectories, and compared these groups to the remainder of the annotated genes (‘None’). No significant differences among these groups were detected when splicing was quantified using exon expression (Fig. 4A, B, gray points). However, when using datasets matched for gene length and chromosomal position (in order to account for background selection dynamics and linkage disequilibrium), the median π of genes without differential exon expression increased (Fig. 4A, black dashed line), to the extent that these genes had significantly higher π than genes with splicing in diapause only (Fig. 4B, black points). For both differential event expression and differential whole-gene expression, we again found that genes with transcriptional differences that were exclusive to diapause progression (Fig. 4A Diap.) exhibited significantly lower π than other gene sets, especially direct-only and ‘None’ genes. Genes that were differentially spliced in both developmental trajectories also had slightly reduced π values to genes with no evidence of splicing, an effect that was amplified by matched resampling (Fig. 4B). Based on posthoc analyses of effect sizes and Dunn’s test statistics we found that the difference among groups was frequently stronger in Luleå than Kullaberg (Fig. 4B, Supplementary Table S9). This same pattern was observed when comparing π among genes with transcriptional variation in the abdomen (Supplementary Fig. S5).

**Fig. 5:**
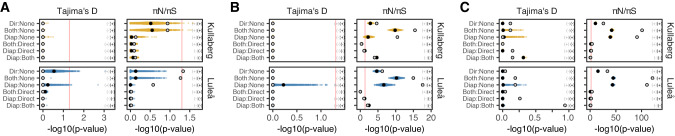
Differences in Tajima’s D and πN/πS among groups of differentially spliced and differentially expressed genes in the head. Genes with **A** differential exon expression, **B** differential event expression, and **C** differential gene expression were grouped according to whether they were differentially spliced or expressed among diapausing timepoints only, both diapausing and direct time points, among direct timepoints only and in genes without transcription variation. Groups were compared with Kruskal–Wallis tests (Supplementary Table S9) with Dunn’s post-hoc pairwise comparisons corrected for multiple tests within each set (Supplementary Table S10). Gray points show *p* values (−log_10_ transformed) of Dunn’s multiple comparisons among full data, while yellow and blue points show *p* values when comparing matched gene sets (*n* = 1000) for each population. Black points summarize means of these comparisons. The direction of the effect is shown in parentheses (gray = full data, black = matched ranges). Red lines represent significance thresholds of 0.05.

Finally, we investigated whether positive selection may have contributed to the low genetic diversity observed in differentially spliced and expressed genes in diapause. Specifically, we investigated whether genes with transcriptional plasticity during diapause exhibited outlier patterns of Tajima’s D compared to neutral expectations (lower under purifying and positive selection), and whether these genes showed different patterns consistent with negative selection using the codon-aware metric π_N_/π_S_ (lower under purifying selection). For all three forms of transcriptional variation, we were unable to consistently detect significant differences in Tajima’s D among diapause-only, direct-only genes and the remaining genes in either the head (Figs. 5A–C, Supplementary Fig. S6) or the abdomen (Supplementary Fig. S7). The exception to this pattern was among differentially expressed genes in the abdomen, where Tajima’s D was lower in the diapause-only genes compared direct-only genes (Supplementary Fig. S9). In contrast, we found that π_N_/π_S_ was significantly lower in genes with transcriptional plasticity through pupal development compared to the remainder of annotated genes, suggesting strong negative selection acting upon these loci. This effect was dampened but still significant when genes were matched by length and relative position and again often stronger in Luleå (Fig. 5A–C, Supplementary Table S10).

## Discussion

*Pieris napi* butterflies exhibit extensive alternative splicing changes, as measured by exon and event expression, through pupal diapause that differ from those seen in direct developing pupae. We used this system to test the more general hypothesis that negative selection to maintain splicing plasticity constrains genetic diversity at the genes involved in maintaining a seasonal polyphenism, expecting to find i) lower π, Tajima’s D, and π_N_/π_S_ in genes with splicing exclusive to either alternative developmental trajectory, ii) the effect would be greater in populations that are under selection to maintain plasticity (i.e., multivoltine populations that alternate between direct and diapausing generations; in this dataset, Kullaberg), and iii) the effect would be unique to, or at least stronger for, genes experiencing differential splicing compared to differential whole-gene expression. We also expected that populations under selection to maintain plasticity might exhibit signatures of positive selection acting upon genes that are differentially spliced between alternative developmental trajectories.

We found instead that the largest genetic constraint (reduced π) was on transcriptional plasticity unique to diapause progression and was highest among genes with splicing unique to direct development or genes that were not differentially spliced through pupal development. This pattern was especially pronounced in the Luleå population, which undergoes diapause every generation, rather than the population with facultative diapause (Kullaberg). Surprisingly, the pattern of low vs. high genetic diversity in uniquely diapause vs. direct genes was also seen in differentially expressed loci. Nevertheless, when we focused on genes with transcriptional plasticity unique to diapause, we still found a stronger effect (i.e., lower diversity) on differentially spliced than differentially expressed genes. Finally, while these patterns were replicated for π in both head and abdomen samples, and supported by π_N_/π_S_, no evidence for positive selection was found using Tajima’s D comparisons.

### Genetic constraints in phenotypically plastic splicing

We predicted that selection to maintain *cis-*regulatory mechanisms involved phenotypic plasticity should result in reduced genetic diversity and possible signatures of directional selection in plastically spliced genes. We also expected this signature to be stronger in populations that rely on developmental plasticity (e.g., annually have direct and diapause generations). This prediction was motivated by evidence that differentially spliced genes between wet and dry morphs of the butterfly *Bicyclus anynana* had decreased π compared both to genes that were not spliced and to those that were alternatively spliced but not significantly different between seasonal morphs (Steward et al. 2022). We considered facultative diapause an ideal polyphenism for testing these predictions because of clear latitudinal variation in plasticity among populations. However, diapause and seasonal polyphenism differ from one another in several key aspects. The most important of these is that the seasonal polyphenism of *B. anynana* allowed for one-to-one comparison of seasonal morphs arising from different developmental trajectories (wet vs. dry adult females). In contrast, diapause progression involves a divergence from and return to direct development, making orthogonal comparisons more complicated. Instead, we characterized differential splicing and differential expression through both developmental trajectories, interpreting unique transcriptional changes within trajectories as seasonal transcriptional plasticity. This produced a detailed investigation of splicing plasticity throughout developmental progression, but may mean we are not targeting the same types of transcriptional changes as in the *B. anynana* study (Steward et al. 2022). This leads us to wonder what a similarly detailed study over development in *B. anynana* would reveal, though we have no predictions as to which morph might experience more genetic constraint.

The observed differences in constraint between the two developmental trajectories was unexpected. The consistent signature of reduced π among genes spliced in diapause suggests that these genes are under strong purifying selection to maintain the diapause phenotype. Moreover, this signature of constraint appears to be slightly stronger in Luleå population, which experiences diapause every generation, although evidence for this decreases in permutations with matched gene sets. While we predicted constraint on plasticity, what we may have identified instead is constraint associated with a critical life history strategy of a temperate insect, complemented by relaxation of selection on plastic gene expression in direct development. Haugen and Gotthard (2015) also found that univoltine populations of the butterfly *Pararge aegeria* in central Sweden exhibit relaxed selection on direct development compared to bivoltine populations further south, optimizing diapause over direct development life history traits. Here, we may be detecting similar patterns at the molecular level, although sampling more *P. napi* populations across the voltinism gradient would be necessary to draw stronger conclusions.

Our conclusions about genetic constraint derive from measurements of π and π_N_/π_S_, whereas results from Tajima’s D showed no differences. While this lack of concordance suggests we should be cautious about potential explanations, it does not exclude purifying selection as a mechanism behind the clear patterns of decreased π in genes with transcriptional plasticity through diapause. Rather, it is likely the strength of purifying selection was not sufficient to disrupt the SFS for this conservative metric (Garrigan et al. 2010). Impressively, estimates of the genome wide SFS using Tajima’s D have had a nearly identical distribution in the two populations. Median Tajima’s D is slightly negative, suggesting that both populations have a similar demographic history of recent expansion (Aris-Brosou and Excoffier 1996) or have experienced historical homogenizing gene flow, possibly influenced by the recent northward expansion of *P. napi* following the recession of the most recent glacial maximum (Porter and Geiger 1995; Schmitt 2007). Both these dynamics could obscure signatures of purifying selection among individual genes. Methods for detecting selection in PoolSeq data, however, are still lacking. Additional tests of selection in these populations that leverage the power of individual genomes, as well as a wider latitudinal sampling of diapause plasticity (voltinism) phenotypes among populations, will be necessary to make stronger conclusions about the relative roles of selection and drift in shaping genetic diversity in these populations.

Although we believe our results provide support for constraints on alternatively spliced genes, they are highly correlational. The consequences of phenotypic plasticity on splicing and the evolution of spliced genes merit further study. One potential route would be to identify changes in alternative splicing under experimental evolution of plasticity, as has been done for whole gene and transcript expression (Leung et al. 2020), and explore differences in π within these genes. Additionally, comparisons of genetic diversity at and around splice junctions, exonic or intronic splicing regulator (ESR, ISR) sites, etc., with regions that are not involved in splice site recognition, would be an effective way to localize the gene-level effects to specific regulatory sequences. Although previous work found that ESRs in alternatively spliced exons tended to have more single nucleotide variants than those in constitutively expressed exons (de Souza et al. 2011), this has never been tested in the context of phenotypic plasticity. Finally, decreased genetic variation within alternatively spliced genes does not necessarily mean that splicing plasticity cannot or will not evolve. Here, we did not explicitly test for genotype by environment interactions in splicing, and thus are unable to identify genetic differences in plasticity that exist within or among populations.

### Neutral and nearly neutral processes and the evolution of alternative splicing

On a mechanistic scale, alternative splicing is a noisy process. Much of this splicing “noise” within an individual occurs either because of errors made by the splicing machinery (Wan and Larson 2018), or due to low frequency mutations affecting splice site recognition or splicing regulatory elements (Pickrell et al. 2010). In this study, we are unable to comment directly on the degree of splicing noise in diapausing and direct developing pupae. Extremely rare splice events and exons with very low coverage across all samples were excluded from our differential expression analyses. Further, differential expression analyses specifically identify exons and events with lower within- than between-group variation and aim to reveal the small subset of genes that are likely to experience regulated changes in splicing through pupal development. Nevertheless, the extent of heritable splicing variation within populations is expected to adhere the same evolutionary forces determining all population variation, that is, fluctuating in frequency within populations due to neutral or nearly neutral conditions (Saudemont et al. 2017). Though *P. napi* population sizes are slightly smaller at northern latitudes, they are generally very large throughout its range (von Schmalensee et al. 2023). This is consistent with our observations that, genome-wide, there were no differences between Kullaberg and Luleå populations, suggesting these populations experience roughly similar nearly neutral evolutionary dynamics.

Genetic variation is expected to experience more drift at differentially spliced genes, as the exons or introns that are only expressed in certain environments, morphs or sexes are predicted to accumulate greater nucleotide diversity when not expressed (Marden 2008; Rogers et al. 2021). Such effects have been observed in systems with sex-biased or morph-biased gene expression (e.g., Purandare et al. 2014; Dutoit et al. 2018 ; although these differences can arise through both neutral and adaptive processes, see Helanterä and Uller 2014). Overall, we did not detect a similar release from negative selection in genes plastically spliced through pupal development, as we observed lower π and πN/πS, especially when splicing was unique to diapausing pupae. This result is consistent with purifying selection within these genes, which we hypothesize is caused by selection on *cis*-regulatory elements such as splicing enhancers and silencers. However, we did find that the strength of this negative selection appears to be lower in the population that only expresses diapause every other generation (Kullaberg), consistent with expectations of more drift at loci not experiencing selection every generation.

### Splicing as a unique axis of transcriptional plasticity

As has previously been found for phenotypically plastic traits (Grantham and Brisson 2018; Steward et al. 2022; Tian and Monteiro 2022), the number of genes demonstrating splicing plasticity was only a fraction of the number of genes with differential whole gene expression. For all comparisons, however, differentially expressed genes represented less than half of the differentially spliced gene sets (Supplementary Table S5). This lack of overlap has previously been associated with functional differences between differentially spliced and differentially expressed genes, for example between benthic and pelagic morphs of arctic char (Jacobs and Elmer 2021) and seasonal morphs of *Bicyclus anynana* butterflies (Steward et al. 2022).

*Pieris napi* differential spicing events were overwhelmingly dominated by SEs and MXEs, which are event types that often evolve from constitutive exons through transition (from constitutive exon to skipped exon) or exon duplication (Wright et al. 2022). It is possible that these processes are more likely in Lepidoptera than processes like exonization, which converts intronic regions to alternatively spliced exons through the insertion of DNA containing splice sites. While lepidopterans have high to moderate levels of repetitive content compared to other insects, the relative proportion of transposable elements (LTRs and DNA transposons) tends to be low (Sproul et al. 2023), which may explain the lack of exonization-associated splice events in *P. napi* and *B. anynana* (Steward et al. 2022; Tian and Monteiro 2022). The consequences of selection on alternatively spliced genes should differ among splice event types (McManus et al. 2014; Steward et al. 2022), partially because splice events differ in their likelihood of producing nonfunctional isoforms. Thus, it would be informative to explore selection on plastically spliced genes in Lepidoptera or other insects that have undergone genome expansions through TE release (e.g., Podsiadlowski et al. 2021).

### Functional consequences of splicing in diapause

Our results support the growing consensus that diapause in butterflies is not a distinct stage, but rather a diversion from the normal direct developmental trajectory (Ragland et al. 2011; Dowle et al. 2020; Pruisscher et al. 2022). Within diapause, the largest splicing differences occurred between day 0 and days 24–144. Much of this transcriptional variation was likely caused by the cold climate conditions at which the diapausing pupae were kept rather than maintenance of diapause itself. For example, cluster ev.A3 was enriched for plasma membrane organization biological processes. Insects that are exposed to low temperatures often compensate through homeoviscous adaptation, where they modify membrane composition to maintain membrane fluidity in cold (Teets and Denlinger 2013). Furthermore, exposure to cold has previously been associated with downregulation of response to hydrogen peroxide (Stuckas et al. 2014), which may correspond to a lowered expectation for oxidative metabolism in cold., further supported by a downregulation of both metabolic processes and regulation of mitochondrial membrane. All three of these biological processes were enriched in cluster ev.H1. Similarly, several clusters with this distinct up or down regulation of exon or event expression in the cold timepoints were enriched for ultradian (within 24 h) rhythmic processes, which may be disrupted in these pupae because they were also kept in the dark during this time.

One mechanism critical to maintenance and termination of diapause is the prothoracicotropic hormone (PTTH)-ecdysone axis, which coordinates the production and secretion of regulatory hormones, including ecdysone. Ecdysone is a steroid that has a key role in initiating the termination of diapause in *P. napi* (Süess et al. 2022). We found several clusters of differentially expressed exons in genes enriched for steroid metabolic processes (specifically the vertebrate steroid glucocorticoid, but this is likely to correspond to insect metabolic pathways like ecdysone signaling), including ex.H4 and ex.A6. These clusters even followed similar splicing patterns: slowly increasing expression of exons through diapause, before dropping expression of these exons in the final diapause timepoint, corresponding to expression in early direct development. We also found several clusters of exon and event expression changes unique to the final timepoint of diapause and days 3 and 6 of direct development, suggesting these are involved in morphological and metabolic changes during pupal metamorphosis. Accordingly, these clusters contained exons and events in both copies of the gene *E75* in the *P. napi* assembly (PieNap_g1361 and PieNap_g1362 in our annotation). *E75* is a signaling gene downstream of ecdysone, different isoforms of which can either provide negative or positive feedback within the PTTH-ecdysone biosynthetic pathway (Li et al. 2016). In sum, our findings show that differential splicing plays a complementary and concerted role in the progression of facultative diapause.

### Supplementary information

Supplementary Material

Table S6

Table S7

Table S8

Table S9

Table S10

## Data Availability

We used archived *Pieirs napi* RNA-sequencing data from Bioproject PRJNA684967. Reads were mapped to the ilPieNapi1 *P. napi* genome assembly from the Darwin Tree of Life Project (GCA_905231885.1). Pool-sequencing data have been archived with the European Nucleotide Archive (PRJEB71016). All shell and R scripts used to generate and analyze the expression data in this study have been archived on GitHub. A final version of the GitHub repository is publicly available through zenodo.org (10.5281/zenodo.10277672).
