# Supplementary material for: Genetic constraints in genes exhibiting splicing plasticity in facultative diapause
Source: Heredity (Edinb). 2024 Jan 30;132(3):142–55. doi: 10.1038/s41437-024-00669-2 (PMC10923799; doi:10.1038/s41437-024-00669-2)
Supplement: Supplementary file 1 — Supplementary Material [file 41437_2024_669_MOESM1_ESM.docx]

**Supplementary materials for “Genetic constraints in genes exhibiting splicing plasticity in facultative diapause”**

Rachel A. Steward, Peter Pruisscher, Kevin T. Roberts, Chris W. Wheat

Table of Contents

[Supplementary methods 1](#_Toc144643444)

[RNA extraction and processing 1](#_Toc144643445)

[In-house annotation 1](#_Toc144643446)

[Poolseq DNA extraction 2](#_Toc144643447)

[Supplementary Tables 3](#_Toc144643448)

[Table S1. 3](#_Toc144643449)

[Table S2. 4](#_Toc144643450)

[Table S3. 5](#_Toc144643451)

[Table S4. 7](#_Toc144643452)

[Table S5. 10](#_Toc144643453)

[Table S6. 10](#_Toc144643454)

[Table S7. 10](#_Toc144643455)

[Table S8. 10](#_Toc144643456)

[Table S9. 11](#_Toc144643457)

[Table S10. 11](#_Toc144643458)

[Supplementary Figures 12](#_Toc144643459)

[Fig. S1. 12](#_Toc144643460)

[Fig. S2. 13](#_Toc144643461)

[Fig. S3. 14](#_Toc144643462)

[Fig. S4. 15](#_Toc144643463)

[Fig. S5. 16](#_Toc144643464)

[Fig. S6. 17](#_Toc144643465)

[Fig. S7. 18](#_Toc144643466)

[Fig. S8. 19](#_Toc144643467)

[Fig. S9. 20](#_Toc144643468)

[Supplementary References 21](#_Toc144643469)

# Supplementary methods

## RNA extraction and processing

Additional details about rearing conditions, sample collection, and RNA extraction can be found in Pruischer et al. (2022). Briefly, mRNA libraries were strand-specific (RF) Illumina TruSeq paired-end reads (2x125bp) with poly-A selection. Library preparation, sequencing and data processing was performed at the National Genomics Infrastructure Sweden (NGI Stockholm). Raw reads were trimmed for adapter sequences using bbduk v. 37 bbduk.sh and bbduk2.sh, distributed by BBtools (<https://jgi.doe.gov/data-and-tools/software-tools/bbtools/>).

## In-house annotation

We used a *Pieris napi* genome assembly from the Darwin Tree of Life Project (GCA_905231885.1). Assembled using Pac-Bio reads from a *P. napi* female (SC_PN_1159, AKA ilPieNapi1) collected from Scotland, UK (Lohse *et al.*, 2021), this is a highly contiguous (N50 = 13M, N90 = 11M) and complete (99.0% complete BUSCOs using the Lepidoptera ODB10 database). The annotation available for this assembly (accessed 7 January 2022) had a large amount of overlapping content – especially unidirectional tandem overlaps on the same strand – between predicted genes, both for coding and noncoding regions of protein-coding genes and for long noncoding RNAs (lncRNA). This can be technically problematic. Many tools for quantifying differential exon usage and differential event expression struggle to assign and distinguish between reads mapping to overlapping transcripts. The degree of overlap was also suspect because overlapping gene features tend to be rare in multicellular eukaryotes, although overlaps between two or more protein-coding genes or between protein-coding genes and lncRNAs appear to be more abundant in eukaryotic genomes than previously believed (Wright *et al.*, 2022). Due to these concerns, we made new annotations for the ilPieNapi1 genome using the BRAKER2 pipeline (v.2.1.5, Lomsadze *et al.*, 2005; Stanke *et al.*, 2006, 2008; Ter-Hovhannisyan *et al.*, 2008; Buchfink *et al.*, 2015; Hoff *et al.*, 2016, 2019; Brůna *et al.*, 2021)

We generated three annotations: protein-informed, RNA-informed and merged. First, we ran BRAKER2 protein mode, using Arthropoda OrthoDB (v.10) reference proteins. We mapped cleaned and trimmed mRNA libraries to the ilPieNapi1 genome using hisat2 (v. 2.2.1, Kim *et al.*, 2019). We specified reverse-strandedness (RF -1), but otherwise used default mapping parameters. After sorting and indexing with Samtools (v. 1.9; Li *et al.*, 2009; Danecek *et al.*, 2021), mapped reads were used as input into BRAKER2 in RNA mode. Finally, we used TSEBRA ( Gabriel *et al.*, 2021) to sort (fix_gtf_ids.py) and merge (tsebra.py) the protein-based and RNA-based annotations. We did not enable UTR annotation in either annotation pipeline, so while there are several 5’ and 3’ UTRs in the BRAKER2 annotations (Table S1), these are not representative of the true abundance of UTRs and subsequent splicing analyses do not fully capture potential variation associated with splicing in the UTRs. We used AGAT (agat_sp_statistics.pl; Dainat *et al.*, 2022) to generate statistics for these three annotations and the Darwin Tree of Life annotation (Table S1). We also used BUSCO (Manni *et al.*, 2021) to assess the completeness of the annotations (Table S2). Through these comparisons, we found that the BRAKER2 protein-informed assembly was the most complete, with the fewest fragmented BUSCOs. The merged annotation had the fewest missing BUSCOs and fewest overlapping genes, but also had an inflated number of single exon genes, fewer total estimated transcripts, and fewer complete BUSCOs. We decided to move forward with the protein-based annotation but acknowledge that the low number of estimated transcripts relative to genes may mean that we may miss some exon- and junction-level variation.

## Poolseq DNA extraction

For each individual, DNA was extracted using a robotic pipeline (cell and tissue DNA kit on a KingFisher Duo Prime purifier, from ThermoFisher Scientific) following standard protocols with added RNAse A to remove contamination. Concentration and purity were quantified via Qubit 2.0 fluorometer and nanodrop (ThermoFisher Scientific). DNA fragmentation was minimal, assessed visually on a 2% agarose gel stained with GelRed. Equal amounts of DNA from each sample were pooled and submitted to SciLifeLab (Stockholm, Sweden) for library preparation and sequencing (Illumina HiSeq), using 150bp paired-end reads with 350bp insert size.

# Supplementary Tables

| Table S1. **Comparison of basic statistics for inhouse and Darwin Tree of Life (DTOL) annotations** for the GCA_905231885.1 *Pieris napi* genome. Inhouse annotations were made using mapped RNA reads (brakerRNA), protein hints (brakerProt), and by merging these two annotations with TSEBRA (brakerMerge). Two DTOL annotations were assessed, one available before March 2023 and the current annotation available on the DTOL data portal. | | | | | |
| --- | --- | --- | --- | --- | --- |
| **Annotation characteristic** | **brakerRNA** | **brakerProt** | **brakerMerge** | **DTOL (pre 3-2022)** | **DTOL (post 3-2022)** |
| Number of genes | 16804 | 16449 | 15088 | 13997 | 13789 |
| Number of mrnas | 19124 | 17894 | 16244 | 30774 | 26707 |
| Number of mrnas with utr both sides | NA | NA | NA | 25574 | 21422 |
| Number of mrnas with at least one utr | 38 | 39 | 16 | 25930 | 22142 |
| Number of cdss | 19124 | 17894 | 16244 | 29998 | 26707 |
| Number of exons | 152102 | 123638 | 104616 | 297270 | 222860 |
| Number of five prime utrs | 20 | 22 | 11 | 25908 | 22038 |
| Number of introns | 121653 | 102259 | 88388 | NA | NA |
| Number of three prime utrs | 18 | 17 | 5 | 25596 | 21526 |
| Number of exon in cds | 152102 | 123638 | 104616 | 252838 | 216274 |
| Number of exon in five prime utr | 20 | 22 | 11 | 51401 | 28356 |
| Number of exon in three prime utr | 18 | 17 | 5 | 43228 | 21526 |
| Number of intron in cds | 132978 | 105744 | 88372 | 222840 | 189576 |
| Number of intron in exon | 132978 | 105744 | 88372 | 266496 | 196153 |
| Number of intron in intron | 106662 | 87790 | 75323 | NA | NA |
| Number gene overlapping | **1986** | **689** | **228** | 4838 | **2570** |
| Number of single exon gene | 2177 | 2706 | 2887 | 1246 | 496 |
| Number of single exon mrna | 2221 | 2806 | 3179 | 1246 | 548 |
| mean mrnas per gene | 1.1 | 1.1 | 1.1 | 2.2 | 1.9 |
| mean exons per mrna | 8 | 6.9 | 6.4 | 9.7 | 8.3 |
| mean five prime utrs per mrna | 0 | 0 | 0 | 0.8 | 0.8 |
| mean introns per mrna | 6.4 | 5.7 | 5.4 | NA | NA |
| mean three prime utrs per mrna | 0 | 0 | 0 | 0.8 | 0.8 |
| mean exons per cds | 8 | 6.9 | 6.4 | 8.4 | 8.1 |
| mean exons per five prime utr | 1 | 1 | 1 | 2 | 1.3 |
| mean exons per three prime utr | 1 | 1 | 1 | 1.7 | 1 |
| mean introns in cdss per mrna | 7 | 5.9 | 5.4 | 7.2 | 7.1 |
| mean introns in exons per mrna | 7 | 5.9 | 5.4 | 8.7 | 7.3 |
| mean introns in introns per mrna | 5.6 | 4.9 | 4.6 | NA | NA |
| Total gene length | 143514864 | 110481963 | 124041888 | 256956212 | 189322560 |
| Total mrna length | 191684655 | 129779344 | 127233238 | 621062157 | 387555492 |
| Total cds length | 30408628 | 27008184 | 22940945 | 51607872 | 41919991 |
| Total exon length | 30464764 | 27090139 | 22976412 | 106928885 | 56743109 |
| Total five prime utr length | 40327 | 62306 | 32251 | 15734215 | 4132299 |
| Total intron length | 151865750 | 99789770 | 104292293 | NA | NA |
| Total three prime utr length | 15809 | 19649 | 3216 | 39425400 | 10690819 |
| Total intron length per cds | 161352869 | 102794949 | 104345198 | 344412082 | 289582841 |
| Total intron length per exon | 161352869 | 102794949 | 104345198 | 514399768 | 331008536 |
| Total intron length per intron | 19460869 | 16545258 | 14010589 | NA | NA |
| mean gene length | 8540 | 6716 | 8221 | 18357 | 13729 |
| mean mrna length | 10023 | 7252 | 7832 | 20181 | 14511 |
| mean cds length | 1590 | 1509 | 1412 | 1720 | 1569 |
| mean exon length | 200 | 219 | 219 | 359 | 254 |
| mean five prime utr length | 2016 | 2832 | 2931 | 607 | 187 |
| mean intron length | 1248 | 975 | 1179 | NA | NA |
| mean three prime utr length | 878 | 1155 | 643 | 1540 | 486 |
| mean cds piece length | 199 | 218 | 219 | 204 | 193 |
| mean five prime utr piece length | 2016 | 2832 | 2931 | 306 | 145 |
| mean three prime utr piece length | 878 | 1155 | 643 | 912 | 496 |
| mean intron in cds length | 1213 | 972 | 1180 | 1545 | 1526 |
| mean intron in exon length | 1213 | 972 | 1180 | 1930 | 1687 |
| mean intron in intron length | 182 | 188 | 186 | NA | NA |
| Longest gene | 163300 | 116077 | 163209 | 293048 | 288269 |
| Longest mrna | 163300 | 116077 | 163209 | 292856 | 288077 |
| Longest cds | 44730 | 58296 | 58296 | 55188 | 44391 |
| Longest exon | 13007 | 14761 | 13007 | 13007 | 13007 |
| Longest five prime utr | 6222 | 9730 | 9730 | 10785 | 781 |
| Longest intron | 69169 | 24540 | 69169 | NA | NA |
| Longest three prime utr | 2100 | 3348 | 1487 | 16886 | 3605 |
| Longest cds piece | 13007 | 14761 | 13007 | 13007 | 13007 |
| Longest five prime utr piece | 6222 | 9730 | 9730 | 10213 | 781 |
| Longest three prime utr piece | 2100 | 3348 | 1487 | 11954 | 3605 |
| Longest intron into cds part | 69170 | 24541 | 69170 | 99973 | 158919 |
| Longest intron into exon part | 69170 | 24541 | 69170 | 99995 | 190864 |
| Longest intron into intron part | 13008 | 14762 | 13008 | NA | NA |
| Shortest gene | 201 | 173 | 173 | 67 | 144 |
| Shortest mrna | 201 | 173 | 173 | 67 | 144 |
| Shortest cds | 62 | 15 | 15 | 225 | 123 |
| Shortest exon | 3 | 3 | 3 | 1 | 1 |
| Shortest five prime utr | 71 | 71 | 71 | 1 | 1 |
| Shortest intron | 42 | 48 | 39 | NA | NA |
| Shortest three prime utr | 50 | 50 | 363 | 1 | 1 |
| Shortest cds piece | 3 | 3 | 3 | 1 | 1 |
| Shortest five prime utr piece | 71 | 71 | 71 | 1 | 1 |
| Shortest three prime utr piece | 50 | 50 | 363 | 1 | NA |
| Shortest intron into cds part | 40 | 35 | 40 | 22 | 22 |
| Shortest intron into exon part | 40 | 35 | 40 | 22 | 22 |
| Shortest intron into intron part | 6 | 13 | 6 | NA | NA |

| Table S2. **Completeness of in-house and Darwin Tree of Life (DTOL) annotations** for the GCA_905231885.1 *Pieris napi* genome assessed using BUSCO (Lepidoptera ODB10). Inhouse annotations were made using mapped RNA reads (brakerRNA), protein hints (brakerProt), and by merging these two annotations with TSEBRA (brakerMerge). Two DTOL annotations were assessed, one available before March 2023 and the current annotation available on the DTOL data portal. | | | | | |
| --- | --- | --- | --- | --- | --- |
| **SCO status in annotation** | **brakerRNA** | **brakerProt** | **brakerMerge** | **DTOL**  **(pre 3-2022)** | **DTOL**  **(post 3-2022)** |
| Complete BUSCOs (C) | 5190 | 5204 | 5191 | 4934 | 4982 |
| Complete and single-copy BUSCOs (S) | 4012 | 4575 | 4915 | 2229 | 2887 |
| Complete and duplicated BUSCOs (D) | 1178 | 629 | 276 | 2705 | 2095 |
| Fragmented BUSCOs (F) | 29 | 16 | 33 | 33 | 40 |
| Missing BUSCOs (M) | 67 | 66 | 62 | 319 | 264 |

| Table S3. **Mapping statistics for *P. napi* RNA samples** mapped with HISAT2 and assessed with Samtools flagstat, reported in millions of reads. | | | | | | | |
| --- | --- | --- | --- | --- | --- | --- | --- |
| **Sample** | **Total** | **Total mapped** | **Multi-mapping** | **Uniquely mapping** | **Uniquely mapping (%)** | **Properly paired (%)** |  |
| P2659_113 | 27.48 | 23.3 | 2 | 21.3 | 83.6 | 69.45 |  |
| P2659_114 | 22.97 | 19.52 | 1.99 | 17.53 | 83.54 | 63.99 |  |
| P2659_115 | 35.98 | 31.9 | 14.72 | 17.18 | 80.82 | 65.91 |  |
| P2659_116 | 20.18 | 16.29 | 2.81 | 13.48 | 77.62 | 62.13 |  |
| P2659_123 | 10.49 | 8.78 | 0.75 | 8.03 | 82.45 | 71.34 |  |
| P2659_124 | 27.92 | 23.63 | 2.01 | 21.62 | 83.46 | 71.36 |  |
| P2659_125 | 37.3 | 31.11 | 2.43 | 28.68 | 82.23 | 69.35 |  |
| P2659_126 | 28.54 | 24.02 | 1.86 | 22.16 | 83.05 | 67.61 |  |
| P2659_129 | 23.3 | 19.12 | 1.29 | 17.83 | 80.98 | 68.62 |  |
| P2659_130 | 24.03 | 19.19 | 1.34 | 17.85 | 78.65 | 64.71 |  |
| P2659_131 | 25.9 | 22.18 | 2.85 | 19.33 | 83.85 | 68.15 |  |
| P2659_132 | 30.41 | 25.93 | 3.43 | 22.5 | 83.38 | 69.6 |  |
| P2659_133 | 26.98 | 22.35 | 4.39 | 17.97 | 79.54 | 67.08 |  |
| P2659_134 | 25.53 | 21.63 | 4.69 | 16.94 | 81.28 | 68.43 |  |
| P2659_135 | 28.25 | 23.67 | 3.99 | 19.68 | 81.13 | 67.03 |  |
| P2659_137 | 27.9 | 23.43 | 4.03 | 19.4 | 81.26 | 67.99 |  |
| P2659_138 | 22.72 | 18.66 | 2.91 | 15.74 | 79.49 | 65.75 |  |
| P2659_139 | 25.99 | 21.1 | 3.58 | 17.52 | 78.16 | 65.01 |  |
| P2659_140 | 24.91 | 20.73 | 3.93 | 16.8 | 80.1 | 65.61 |  |
| P2659_141 | 23.31 | 19.15 | 2.56 | 16.58 | 79.94 | 67.7 |  |
| P2659_142 | 31.36 | 26.34 | 3.62 | 22.72 | 81.92 | 69.76 |  |
| P2659_143 | 34.2 | 28.77 | 3.86 | 24.91 | 82.11 | 69.63 |  |
| P2659_144 | 31.08 | 26.12 | 4.14 | 21.98 | 81.56 | 68.65 |  |
| P2659_146 | 34.25 | 28.42 | 4.59 | 23.83 | 80.33 | 67.94 |  |
| P2659_147 | 28.33 | 23.92 | 4.08 | 19.84 | 81.83 | 68.35 |  |
| P2659_148 | 25.09 | 20.66 | 3.36 | 17.3 | 79.63 | 66.66 |  |
| P2659_149 | 25.12 | 21.02 | 2.95 | 18.07 | 81.5 | 70.35 |  |
| P2659_150 | 33.62 | 28.35 | 4.34 | 24.01 | 82.01 | 67.78 |  |
| P2659_151 | 28.96 | 24.87 | 3.57 | 21.3 | 83.89 | 70.77 |  |
| P2659_152 | 27.18 | 23.08 | 2.99 | 20.09 | 83.07 | 69.31 |  |
| P2659_153 | 28.16 | 24.24 | 4.43 | 19.81 | 83.49 | 71.78 |  |
| P2659_154 | 28.43 | 23.72 | 3.36 | 20.36 | 81.22 | 70.32 |  |
| P2659_155 | 25.77 | 21.35 | 3.37 | 17.97 | 80.27 | 67.88 |  |
| P2659_156 | 27.43 | 23.06 | 4.1 | 18.97 | 81.27 | 70.74 |  |
| P2659_157 | 28.61 | 23.59 | 2.77 | 20.82 | 80.58 | 68.09 |  |
| P2659_158 | 33.13 | 28.48 | 2.91 | 25.57 | 84.62 | 73.28 |  |
| P2659_159 | 34.2 | 29.2 | 3.65 | 25.55 | 83.64 | 71.22 |  |
| P2659_160 | 32.57 | 28.22 | 3.32 | 24.9 | 85.12 | 73.47 |  |
| P2659_161 | 25.42 | 21.29 | 1.94 | 19.35 | 82.4 | 70.1 |  |
| P2659_162 | 30.14 | 25.15 | 2.25 | 22.9 | 82.12 | 70.18 |  |
| P2659_163 | 18.58 | 15.35 | 1.14 | 14.21 | 81.49 | 68.83 |  |
| P2659_164 | 32.89 | 27.9 | 2.13 | 25.76 | 83.77 | 70.36 |  |
| P2659_165 | 39.92 | 34.02 | 5.08 | 28.94 | 83.06 | 69.79 |  |
| P2659_166 | 38.79 | 33.61 | 4.96 | 28.65 | 84.7 | 71.2 |  |
| P2659_167 | 31.68 | 26.99 | 3.29 | 23.7 | 83.46 | 69.51 |  |
| P2659_168 | 33.62 | 29.24 | 3.43 | 25.81 | 85.49 | 70.76 |  |
| P2659_169 | 42.2 | 36.68 | 5.35 | 31.33 | 85 | 69.63 |  |
| P2659_170 | 35.24 | 30.49 | 4.09 | 26.4 | 84.76 | 72.61 |  |
| P2659_171 | 34.11 | 28.97 | 4.35 | 24.62 | 82.74 | 67.61 |  |
| P2659_172 | 35.96 | 30.7 | 4.19 | 26.52 | 83.46 | 69.79 |  |
| P2659_173 | 27.11 | 23.72 | 2.56 | 21.16 | 86.21 | 74.52 |  |
| P2659_174 | 31.26 | 27.09 | 3.17 | 23.92 | 85.14 | 72.05 |  |
| P2659_175 | 29.4 | 25.42 | 2.64 | 22.78 | 85.11 | 71.43 |  |
| P2659_176 | 33.7 | 29.11 | 3.3 | 25.82 | 84.92 | 71.84 |  |
| P2659_177 | 31.86 | 27.98 | 3.51 | 24.47 | 86.33 | 73.91 |  |
| P2659_178 | 30.64 | 26.78 | 3.24 | 23.54 | 85.91 | 73.51 |  |
| P2659_180 | 31.84 | 27.81 | 3.43 | 24.38 | 85.82 | 73.34 |  |
| P2659_181 | 18.77 | 16.41 | 1.72 | 14.69 | 86.17 | 74.18 |  |
| P2659_182 | 30.88 | 26.93 | 3.41 | 23.52 | 85.64 | 71.03 |  |
| P2659_183 | 32.79 | 28.87 | 3.14 | 25.73 | 86.79 | 74.69 |  |
| P2659_184 | 25.38 | 22.35 | 2.54 | 19.82 | 86.75 | 74.44 |  |
| P2659_185 | 26.22 | 23.07 | 3.24 | 19.84 | 86.32 | 73.97 |  |
| P2659_186 | 32.76 | 28.84 | 3.56 | 25.28 | 86.55 | 74.69 |  |
| P2659_187 | 28.49 | 24.74 | 2.87 | 21.87 | 85.38 | 70.16 |  |
| P2659_188 | 34.56 | 30.36 | 4.2 | 26.17 | 86.19 | 75.32 |  |
| P2659_189 | 24.63 | 21.14 | 1.7 | 19.44 | 84.8 | 73.8 |  |
| P2659_190 | 31.46 | 27.23 | 2.1 | 25.13 | 85.59 | 73.28 |  |
| P2659_191 | 30.79 | 26.79 | 2.34 | 24.45 | 85.94 | 73.69 |  |
| P2659_192 | 26.75 | 23.39 | 1.8 | 21.59 | 86.51 | 73.26 |  |
| P2659_193 | 34.66 | 28.51 | 2.23 | 26.28 | 81.04 | 68.99 |  |
| P2659_194 | 23.47 | 18.8 | 1.32 | 17.48 | 78.92 | 66.4 |  |
| P2659_195 | 27.37 | 21.77 | 1.73 | 20.04 | 78.17 | 64.21 |  |
| P2659_196 | 28.07 | 22.79 | 1.7 | 21.08 | 79.97 | 67.03 |  |
| P2659_201 | 29.91 | 23.81 | 3.76 | 20.05 | 76.68 | 65.34 |  |
| P2659_202 | 29.01 | 24 | 3.76 | 20.25 | 80.18 | 68.3 |  |
| P2659_203 | 24.82 | 20.75 | 2.71 | 18.04 | 81.6 | 69.96 |  |
| P2659_205 | 29.04 | 24 | 3.72 | 20.28 | 80.07 | 68.66 |  |
| P2659_206 | 23.78 | 19.68 | 2.67 | 17.01 | 80.57 | 68.16 |  |
| P2659_207 | 34.61 | 28.08 | 2.11 | 25.97 | 79.91 | 67.54 |  |
| P2659_208 | 34.9 | 29.33 | 2.38 | 26.94 | 82.85 | 70.93 |  |
| P2659_209 | 28.37 | 23.64 | 2.96 | 20.68 | 81.41 | 69.27 |  |
| P2659_210 | 28.32 | 24.14 | 2.54 | 21.6 | 83.78 | 72.64 |  |
| P2659_211 | 31.68 | 26.74 | 2.51 | 24.23 | 83.06 | 70.64 |  |
| P2659_212 | 28.95 | 24.12 | 1.94 | 22.18 | 82.13 | 68.44 |  |
| P2659_213 | 35.17 | 29.67 | 2.67 | 27 | 83.07 | 70.79 |  |
| P2659_214 | 31.74 | 26.85 | 2.31 | 24.54 | 83.39 | 70.53 |  |
| P2659_215 | 34.02 | 26.92 | 2.82 | 24.1 | 77.23 | 64.56 |  |
| P2659_216 | 34.77 | 27.44 | 2.76 | 24.68 | 77.11 | 66.17 |  |
| P2659_301 | 27.29 | 21.42 | 2.29 | 19.13 | 76.51 | 68.11 |  |
| P3401_102 | 36.54 | 31.49 | 2.34 | 29.15 | 85.22 | 77.72 |  |
| P3401_103 | 40.19 | 34.36 | 2.85 | 31.51 | 84.38 | 72.34 |  |
| P3401_104 | 44.04 | 38.33 | 5.62 | 32.71 | 85.13 | 76.68 |  |
| P3401_105 | 52.18 | 46.05 | 6.37 | 39.69 | 86.62 | 74.55 |  |
| P3401_106 | 48.9 | 44.11 | 5.04 | 39.08 | 89.09 | 77.19 |  |
| P3401_107 | 50.68 | 42.97 | 4.41 | 38.55 | 83.34 | 71.12 |  |

| Table S4. **Fisher’s exact tests for over- or underrepresentation of event types** compared to expectations between groups 1 and 2, representing different timepoints and developmental trajectories (Dia = diapause, Dir = direct development. Expected significant splice events were estimated based on the total splice events and total significant splice events within each comparison. Note the non-independence of our estimates of expected significant events violates the assumptions of the Fisher’s exact test, however the results illustrate general over- and under-representation of events for each comparison. P-values were adjusted using the Benjamini-Hochberg correction for five tests within each comparison. | | | | | | | | | | |
| --- | --- | --- | --- | --- | --- | --- | --- | --- | --- | --- |
| **Tissue** | **Group 1** | **Group 2** | **Event** | **Splice events** | **Genes** | **Events per gene** | **Sig. splice events** | **Expected sig. events** | **P-value** | **Adj. P-value** |
| Head | Dia000 | Dia003 | A3SS | 617 | 409 | 1.51 | 46 | 53 | 5.30E-01 | 1.00E+00 |
| Head | Dia000 | Dia003 | A5SS | 451 | 334 | 1.35 | 56 | 39 | 8.22E-02 | 4.11E-01 |
| Head | Dia000 | Dia003 | SE | 2092 | 701 | 2.98 | 332 | 179 | 5.06E-13 | **2.53E-12** |
| Head | Dia000 | Dia003 | IR | 538 | 382 | 1.41 | 28 | 46 | 3.99E-02 | 1.99E-01 |
| Head | Dia000 | Dia003 | MXE | 4070 | 1758 | 2.32 | 203 | 348 | 1.69E-10 | **8.45E-10** |
| Head | Dia000 | Dia006 | A3SS | 665 | 436 | 1.53 | 120 | 88 | 1.91E-02 | **9.55E-02** |
| Head | Dia000 | Dia006 | A5SS | 489 | 358 | 1.37 | 93 | 65 | 1.88E-02 | **9.40E-02** |
| Head | Dia000 | Dia006 | SE | 2131 | 726 | 2.94 | 491 | 281 | 6.54E-17 | **3.27E-16** |
| Head | Dia000 | Dia006 | IR | 549 | 385 | 1.43 | 76 | 72 | 7.91E-01 | 1.00E+00 |
| Head | Dia000 | Dia006 | MXE | 4103 | 1758 | 2.33 | 267 | 541 | 1.89E-24 | **9.45E-24** |
| Head | Dia000 | Dia024 | A3SS | 686 | 450 | 1.52 | 221 | 157 | 1.37E-04 | **6.85E-04** |
| Head | Dia000 | Dia024 | A5SS | 524 | 367 | 1.43 | 189 | 120 | 3.83E-06 | **1.91E-05** |
| Head | Dia000 | Dia024 | SE | 2210 | 704 | 3.14 | 789 | 507 | 1.25E-20 | **6.25E-20** |
| Head | Dia000 | Dia024 | IR | 855 | 580 | 1.47 | 215 | 196 | 3.08E-01 | 1.00E+00 |
| Head | Dia000 | Dia024 | MXE | 4073 | 1707 | 2.39 | 502 | 935 | 1.30E-36 | **6.50E-36** |
| Head | Dia000 | Dia114 | A3SS | 853 | 549 | 1.55 | 325 | 250 | 1.48E-04 | **7.40E-04** |
| Head | Dia000 | Dia114 | A5SS | 653 | 442 | 1.48 | 286 | 191 | 6.13E-08 | **3.07E-07** |
| Head | Dia000 | Dia114 | SE | 2677 | 813 | 3.29 | 1176 | 785 | 1.39E-28 | **6.95E-28** |
| Head | Dia000 | Dia114 | IR | 772 | 533 | 1.45 | 232 | 226 | 7.81E-01 | 1.00E+00 |
| Head | Dia000 | Dia114 | MXE | 4320 | 1768 | 2.44 | 700 | 1266 | 3.79E-48 | **1.89E-47** |
| Head | Dia000 | Dia144 | A3SS | 971 | 614 | 1.58 | 362 | 266 | 3.93E-06 | **1.96E-05** |
| Head | Dia000 | Dia144 | A5SS | 708 | 499 | 1.42 | 300 | 194 | 4.34E-09 | **2.17E-08** |
| Head | Dia000 | Dia144 | SE | 2837 | 850 | 3.34 | 1114 | 778 | 3.33E-21 | **1.67E-20** |
| Head | Dia000 | Dia144 | IR | 892 | 598 | 1.49 | 253 | 245 | 7.12E-01 | 1.00E+00 |
| Head | Dia000 | Dia144 | MXE | 4442 | 1815 | 2.45 | 673 | 1219 | 8.67E-46 | **4.33E-45** |
| Head | Dia000 | Dia155 | A3SS | 650 | 442 | 1.47 | 42 | 79 | 5.44E-04 | **2.72E-03** |
| Head | Dia000 | Dia155 | A5SS | 454 | 357 | 1.27 | 57 | 55 | 9.20E-01 | 1.00E+00 |
| Head | Dia000 | Dia155 | SE | 2169 | 716 | 3.03 | 455 | 262 | 3.07E-15 | **1.54E-14** |
| Head | Dia000 | Dia155 | IR | 535 | 390 | 1.37 | 26 | 65 | 2.44E-05 | **1.22E-04** |
| Head | Dia000 | Dia155 | MXE | 4120 | 1777 | 2.32 | 379 | 498 | 2.44E-05 | **1.22E-04** |
| Head | Dia003 | Dia006 | A3SS | 673 | 452 | 1.49 | 32 | 36 | 7.09E-01 | 1.00E+00 |
| Head | Dia003 | Dia006 | A5SS | 491 | 362 | 1.36 | 44 | 26 | 3.43E-02 | 1.71E-01 |
| Head | Dia003 | Dia006 | SE | 2196 | 724 | 3.03 | 213 | 117 | 4.44E-08 | **2.22E-07** |
| Head | Dia003 | Dia006 | IR | 541 | 387 | 1.40 | 23 | 29 | 4.78E-01 | 1.00E+00 |
| Head | Dia003 | Dia006 | MXE | 4172 | 1763 | 2.37 | 118 | 222 | 9.17E-09 | **4.59E-08** |
| Head | Dia006 | Dia024 | A3SS | 745 | 480 | 1.55 | 190 | 154 | 3.13E-02 | 1.57E-01 |
| Head | Dia006 | Dia024 | A5SS | 576 | 399 | 1.44 | 175 | 119 | 1.96E-04 | **9.80E-04** |
| Head | Dia006 | Dia024 | SE | 2235 | 708 | 3.16 | 692 | 462 | 4.28E-15 | **2.14E-14** |
| Head | Dia006 | Dia024 | IR | 856 | 573 | 1.49 | 238 | 177 | 7.04E-04 | **3.52E-03** |
| Head | Dia006 | Dia024 | MXE | 4194 | 1709 | 2.45 | 483 | 866 | 3.36E-30 | **1.68E-29** |
| Head | Dia024 | Dia114 | A3SS | 845 | 530 | 1.59 | 98 | 74 | 6.40E-02 | 3.20E-01 |
| Head | Dia024 | Dia114 | A5SS | 588 | 403 | 1.46 | 60 | 52 | 4.87E-01 | 1.00E+00 |
| Head | Dia024 | Dia114 | SE | 2554 | 748 | 3.41 | 343 | 225 | 1.76E-07 | **8.80E-07** |
| Head | Dia024 | Dia114 | IR | 647 | 450 | 1.44 | 38 | 57 | 5.46E-02 | 2.73E-01 |
| Head | Dia024 | Dia114 | MXE | 4022 | 1656 | 2.43 | 223 | 354 | 1.71E-08 | **8.55E-08** |
| Head | Dia114 | Dia144 | A3SS | 965 | 613 | 1.57 | 19 | 20 | 1.00E+00 | 1.00E+00 |
| Head | Dia114 | Dia144 | A5SS | 739 | 496 | 1.49 | 18 | 15 | 7.25E-01 | 1.00E+00 |
| Head | Dia114 | Dia144 | SE | 2805 | 820 | 3.42 | 92 | 59 | 8.09E-03 | **4.05E-02** |
| Head | Dia114 | Dia144 | IR | 899 | 598 | 1.50 | 28 | 19 | 2.37E-01 | 1.00E+00 |
| Head | Dia114 | Dia144 | MXE | 4349 | 1769 | 2.46 | 47 | 91 | 2.01E-04 | **1.01E-03** |
| Head | Dia144 | Dia155 | A3SS | 1069 | 682 | 1.57 | 409 | 329 | 3.23E-04 | **1.62E-03** |
| Head | Dia144 | Dia155 | A5SS | 758 | 540 | 1.40 | 348 | 233 | 1.57E-09 | **7.85E-09** |
| Head | Dia144 | Dia155 | SE | 3066 | 910 | 3.37 | 1336 | 944 | 4.15E-25 | **2.08E-24** |
| Head | Dia144 | Dia155 | IR | 904 | 602 | 1.50 | 275 | 278 | 9.19E-01 | 1.00E+00 |
| Head | Dia144 | Dia155 | MXE | 4549 | 1847 | 2.46 | 818 | 1401 | 3.19E-46 | **1.60E-45** |
| Head | Dir000 | Dia000 | A3SS | 513 | 344 | 1.49 | 12 | 17 | 4.52E-01 | 1.00E+00 |
| Head | Dir000 | Dia000 | A5SS | 369 | 280 | 1.32 | 8 | 12 | 4.97E-01 | 1.00E+00 |
| Head | Dir000 | Dia000 | SE | 1781 | 622 | 2.86 | 108 | 58 | 8.82E-05 | **4.41E-04** |
| Head | Dir000 | Dia000 | IR | 487 | 356 | 1.37 | 5 | 16 | 2.50E-02 | 1.25E-01 |
| Head | Dir000 | Dia000 | MXE | 3869 | 1704 | 2.27 | 97 | 127 | 4.90E-02 | 2.45E-01 |
| Head | Dir000 | Dir003 | A3SS | 620 | 441 | 1.41 | 28 | 58 | 1.08E-03 | **5.40E-03** |
| Head | Dir000 | Dir003 | A5SS | 457 | 350 | 1.31 | 42 | 43 | 1.00E+00 | 1.00E+00 |
| Head | Dir000 | Dir003 | SE | 2140 | 746 | 2.87 | 332 | 201 | 1.50E-09 | **7.50E-09** |
| Head | Dir000 | Dir003 | IR | 613 | 450 | 1.36 | 19 | 57 | 7.94E-06 | **3.97E-05** |
| Head | Dir000 | Dir003 | MXE | 4025 | 1765 | 2.28 | 315 | 377 | 1.52E-02 | 7.60E-02 |
| Head | Dir000 | Dir006 | A3SS | 555 | 392 | 1.42 | 58 | 90 | 6.05E-03 | **3.03E-02** |
| Head | Dir000 | Dir006 | A5SS | 425 | 310 | 1.37 | 74 | 69 | 7.14E-01 | 1.00E+00 |
| Head | Dir000 | Dir006 | SE | 2166 | 732 | 2.96 | 608 | 353 | 1.04E-20 | **5.20E-20** |
| Head | Dir000 | Dir006 | IR | 496 | 355 | 1.40 | 31 | 81 | 6.10E-07 | **3.05E-06** |
| Head | Dir000 | Dir006 | MXE | 4010 | 1734 | 2.31 | 475 | 653 | 1.23E-08 | **6.15E-08** |
| Head | Dir003 | Dir006 | A3SS | 671 | 472 | 1.42 | 43 | 65 | 3.47E-02 | 1.74E-01 |
| Head | Dir003 | Dir006 | A5SS | 504 | 377 | 1.34 | 57 | 49 | 4.72E-01 | 1.00E+00 |
| Head | Dir003 | Dir006 | SE | 2417 | 801 | 3.02 | 423 | 235 | 3.03E-15 | **1.52E-14** |
| Head | Dir003 | Dir006 | IR | 612 | 437 | 1.40 | 27 | 59 | 4.65E-04 | **2.33E-03** |
| Head | Dir003 | Dir006 | MXE | 4047 | 1762 | 2.30 | 252 | 393 | 8.08E-09 | **4.04E-08** |
| Abdomen | Dia000 | Dia003 | A3SS | 349 | 234 | 1.49 | 8 | 20 | 3.21E-02 | 1.60E-01 |
| Abdomen | Dia000 | Dia003 | A5SS | 253 | 175 | 1.45 | 15 | 15 | 1.00E+00 | 1.00E+00 |
| Abdomen | Dia000 | Dia003 | SE | 1270 | 404 | 3.14 | 150 | 74 | 1.20E-07 | **6.00E-07** |
| Abdomen | Dia000 | Dia003 | IR | 249 | 172 | 1.45 | 16 | 14 | 8.51E-01 | 1.00E+00 |
| Abdomen | Dia000 | Dia003 | MXE | 3243 | 1419 | 2.29 | 123 | 189 | 1.54E-04 | **7.70E-04** |
| Abdomen | Dia000 | Dia006 | A3SS | 388 | 257 | 1.51 | 28 | 31 | 7.87E-01 | 1.00E+00 |
| Abdomen | Dia000 | Dia006 | A5SS | 288 | 209 | 1.38 | 22 | 23 | 1.00E+00 | 1.00E+00 |
| Abdomen | Dia000 | Dia006 | SE | 1446 | 442 | 3.27 | 208 | 115 | 4.66E-08 | **2.33E-07** |
| Abdomen | Dia000 | Dia006 | IR | 263 | 180 | 1.46 | 14 | 21 | 2.94E-01 | 1.00E+00 |
| Abdomen | Dia000 | Dia006 | MXE | 3413 | 1463 | 2.33 | 188 | 271 | 7.12E-05 | **3.56E-04** |
| Abdomen | Dia000 | Dia024 | A3SS | 505 | 330 | 1.53 | 124 | 85 | 3.10E-03 | **1.55E-02** |
| Abdomen | Dia000 | Dia024 | A5SS | 360 | 247 | 1.46 | 101 | 61 | 4.77E-04 | **2.39E-03** |
| Abdomen | Dia000 | Dia024 | SE | 1809 | 526 | 3.44 | 496 | 306 | 3.13E-14 | **1.57E-13** |
| Abdomen | Dia000 | Dia024 | IR | 387 | 264 | 1.47 | 77 | 65 | 3.07E-01 | 1.00E+00 |
| Abdomen | Dia000 | Dia024 | MXE | 3536 | 1468 | 2.41 | 317 | 598 | 1.69E-23 | **8.45E-23** |
| Abdomen | Dia000 | Dia114 | A3SS | 614 | 408 | 1.50 | 200 | 136 | 5.32E-05 | **2.66E-04** |
| Abdomen | Dia000 | Dia114 | A5SS | 456 | 313 | 1.46 | 179 | 101 | 2.79E-08 | **1.40E-07** |
| Abdomen | Dia000 | Dia114 | SE | 1949 | 575 | 3.39 | 703 | 433 | 1.81E-21 | **9.05E-21** |
| Abdomen | Dia000 | Dia114 | IR | 481 | 352 | 1.37 | 114 | 107 | 6.46E-01 | 1.00E+00 |
| Abdomen | Dia000 | Dia114 | MXE | 3705 | 1551 | 2.39 | 403 | 822 | 1.10E-39 | **5.50E-39** |
| Abdomen | Dia000 | Dia144 | A3SS | 588 | 383 | 1.54 | 186 | 120 | 1.48E-05 | **7.40E-05** |
| Abdomen | Dia000 | Dia144 | A5SS | 421 | 296 | 1.42 | 156 | 86 | 1.28E-07 | **6.40E-07** |
| Abdomen | Dia000 | Dia144 | SE | 1898 | 565 | 3.36 | 560 | 388 | 1.32E-10 | **6.60E-10** |
| Abdomen | Dia000 | Dia144 | IR | 517 | 371 | 1.39 | 137 | 106 | 2.77E-02 | 1.38E-01 |
| Abdomen | Dia000 | Dia144 | MXE | 3664 | 1545 | 2.37 | 410 | 749 | 1.32E-27 | **6.60E-27** |
| Abdomen | Dia000 | Dia155 | A3SS | 505 | 358 | 1.41 | 70 | 72 | 9.28E-01 | 1.00E+00 |
| Abdomen | Dia000 | Dia155 | A5SS | 365 | 281 | 1.30 | 66 | 52 | 1.91E-01 | 9.55E-01 |
| Abdomen | Dia000 | Dia155 | SE | 1855 | 590 | 3.14 | 445 | 263 | 3.05E-14 | **1.53E-13** |
| Abdomen | Dia000 | Dia155 | IR | 411 | 294 | 1.40 | 49 | 58 | 4.07E-01 | 1.00E+00 |
| Abdomen | Dia000 | Dia155 | MXE | 3876 | 1666 | 2.33 | 365 | 550 | 8.24E-11 | **4.12E-10** |
| Abdomen | Dia003 | Dia006 | A3SS | 467 | 296 | 1.58 | 26 | 18 | 2.80E-01 | 1.00E+00 |
| Abdomen | Dia003 | Dia006 | A5SS | 362 | 269 | 1.35 | 30 | 14 | 1.87E-02 | 9.35E-02 |
| Abdomen | Dia003 | Dia006 | SE | 1593 | 500 | 3.19 | 87 | 62 | 4.37E-02 | 2.19E-01 |
| Abdomen | Dia003 | Dia006 | IR | 389 | 273 | 1.42 | 11 | 15 | 5.50E-01 | 1.00E+00 |
| Abdomen | Dia003 | Dia006 | MXE | 3781 | 1589 | 2.38 | 101 | 146 | 4.33E-03 | 2.17E-02 |
| Abdomen | Dia006 | Dia024 | A3SS | 661 | 396 | 1.67 | 169 | 114 | 2.84E-04 | **1.42E-03** |
| Abdomen | Dia006 | Dia024 | A5SS | 481 | 339 | 1.42 | 136 | 83 | 6.01E-05 | **3.01E-04** |
| Abdomen | Dia006 | Dia024 | SE | 2141 | 617 | 3.47 | 587 | 369 | 1.34E-15 | **6.70E-15** |
| Abdomen | Dia006 | Dia024 | IR | 613 | 417 | 1.47 | 160 | 106 | 2.32E-04 | **1.16E-03** |
| Abdomen | Dia006 | Dia024 | MXE | 4041 | 1629 | 2.48 | 317 | 697 | 7.23E-38 | **3.62E-37** |
| Abdomen | Dia024 | Dia114 | A3SS | 774 | 481 | 1.61 | 66 | 65 | 1.00E+00 | 1.00E+00 |
| Abdomen | Dia024 | Dia114 | A5SS | 580 | 404 | 1.44 | 39 | 49 | 3.18E-01 | 1.00E+00 |
| Abdomen | Dia024 | Dia114 | SE | 2371 | 686 | 3.46 | 321 | 200 | 2.22E-08 | **1.11E-07** |
| Abdomen | Dia024 | Dia114 | IR | 759 | 524 | 1.45 | 52 | 64 | 2.88E-01 | 1.00E+00 |
| Abdomen | Dia024 | Dia114 | MXE | 4129 | 1672 | 2.47 | 249 | 349 | 2.53E-05 | **1.26E-04** |
| Abdomen | Dia114 | Dia144 | A3SS | 797 | 500 | 1.59 | 28 | 26 | 8.90E-01 | 1.00E+00 |
| Abdomen | Dia114 | Dia144 | A5SS | 609 | 417 | 1.46 | 22 | 20 | 8.75E-01 | 1.00E+00 |
| Abdomen | Dia114 | Dia144 | SE | 2329 | 690 | 3.38 | 127 | 75 | 2.27E-04 | **1.14E-03** |
| Abdomen | Dia114 | Dia144 | IR | 794 | 548 | 1.45 | 22 | 26 | 6.61E-01 | 1.00E+00 |
| Abdomen | Dia114 | Dia144 | MXE | 4078 | 1669 | 2.44 | 80 | 132 | 3.64E-04 | **1.82E-03** |
| Abdomen | Dia144 | Dia155 | A3SS | 844 | 541 | 1.56 | 269 | 192 | 3.20E-05 | **1.60E-04** |
| Abdomen | Dia144 | Dia155 | A5SS | 643 | 456 | 1.41 | 227 | 146 | 8.22E-07 | **4.11E-06** |
| Abdomen | Dia144 | Dia155 | SE | 2566 | 771 | 3.33 | 884 | 583 | 1.50E-20 | **7.50E-20** |
| Abdomen | Dia144 | Dia155 | IR | 829 | 568 | 1.46 | 200 | 188 | 5.23E-01 | 1.00E+00 |
| Abdomen | Dia144 | Dia155 | MXE | 4314 | 1772 | 2.43 | 510 | 980 | 2.95E-41 | **1.48E-40** |
| Abdomen | Dir000 | Dia000 | A3SS | 267 | 190 | 1.41 | 8 | 16 | 1.42E-01 | 7.10E-01 |
| Abdomen | Dir000 | Dia000 | A5SS | 225 | 164 | 1.37 | 12 | 14 | 8.40E-01 | 1.00E+00 |
| Abdomen | Dir000 | Dia000 | SE | 1033 | 317 | 3.26 | 90 | 63 | 2.86E-02 | 1.43E-01 |
| Abdomen | Dir000 | Dia000 | IR | 349 | 255 | 1.37 | 22 | 21 | 1.00E+00 | 1.00E+00 |
| Abdomen | Dir000 | Dia000 | MXE | 3257 | 1371 | 2.38 | 183 | 200 | 3.99E-01 | 1.00E+00 |
| Abdomen | Dir000 | Dir003 | A3SS | 466 | 320 | 1.46 | 29 | 31 | 8.94E-01 | 1.00E+00 |
| Abdomen | Dir000 | Dir003 | A5SS | 355 | 266 | 1.33 | 40 | 24 | 4.86E-02 | 2.43E-01 |
| Abdomen | Dir000 | Dir003 | SE | 1700 | 547 | 3.11 | 195 | 115 | 2.25E-06 | **1.13E-05** |
| Abdomen | Dir000 | Dir003 | IR | 522 | 377 | 1.38 | 13 | 35 | 1.64E-03 | **8.20E-03** |
| Abdomen | Dir000 | Dir003 | MXE | 3973 | 1631 | 2.44 | 196 | 268 | 6.68E-04 | **3.34E-03** |
| Abdomen | Dir000 | Dir006 | A3SS | 416 | 290 | 1.43 | 48 | 54 | 5.97E-01 | 1.00E+00 |
| Abdomen | Dir000 | Dir006 | A5SS | 343 | 258 | 1.33 | 52 | 44 | 4.41E-01 | 1.00E+00 |
| Abdomen | Dir000 | Dir006 | SE | 1558 | 510 | 3.05 | 338 | 202 | 1.39E-10 | **6.95E-10** |
| Abdomen | Dir000 | Dir006 | IR | 585 | 436 | 1.34 | 37 | 76 | 1.49E-04 | **7.45E-04** |
| Abdomen | Dir000 | Dir006 | MXE | 3840 | 1595 | 2.41 | 397 | 497 | 4.23E-04 | **2.11E-03** |
| Abdomen | Dir003 | Dir006 | A3SS |  | 344 | 1.45 | 53 | 62 | 4.28E-01 | 1.00E+00 |
| Abdomen | Dir003 | Dir006 | A5SS | 417 | 327 | 1.28 | 50 | 51 | 1.00E+00 | 1.00E+00 |
| Abdomen | Dir003 | Dir006 | SE | 1755 | 607 | 2.89 | 366 | 216 | 1.10E-11 | **5.50E-11** |
| Abdomen | Dir003 | Dir006 | IR | 667 | 482 | 1.38 | 49 | 82 | 3.12E-03 | 1.56E-02 |
| Abdomen | Dir003 | Dir006 | MXE | 3879 | 1668 | 2.33 | 372 | 478 | 1.33E-04 | **6.65E-04** |

| Table S5. **Number of genes (sets) with differential exon, event or whole gene expression** between groups 1 and 2, representing different timepoints and developmental trajectories (Dia = diapause, Dir = direct development), and the number of genes shared between these sources of transcriptional variation (intersections). | | | | | | | | | |
| --- | --- | --- | --- | --- | --- | --- | --- | --- | --- |
| **Group1** | **Group2** | **Tissue** | **Sets** | | | **Intersections** | | | |
|  |  |  | **Exon** | **Event** | **Gene** | **Exon-Event** | **Exon-Gene** | **Event-Gene** | **Exon-Event-Gene** |
| Dir000 | Dia000 | Head | 0 | 95 | 348 | 0 | 0 | 9 | 0 |
| Dir000 | Dir003 | Head | 32 | 241 | 1334 | 8 | 3 | 42 | 10 |
| Dir000 | Dir006 | Head | 86 | 332 | 4257 | 12 | 30 | 159 | 26 |
| Dir003 | Dir006 | Head | 30 | 273 | 2534 | 1 | 10 | 102 | 11 |
| Dia000 | Dia003 | Head | 5 | 239 | 1189 | 0 | 1 | 49 | 0 |
| Dia000 | Dia006 | Head | 22 | 406 | 3091 | 2 | 9 | 149 | 6 |
| Dia000 | Dia024 | Head | 36 | 670 | 3884 | 9 | 8 | 312 | 11 |
| Dia000 | Dia114 | Head | 44 | 848 | 3972 | 12 | 5 | 389 | 21 |
| Dia000 | Dia144 | Head | 52 | 933 | 3928 | 14 | 5 | 411 | 25 |
| Dia000 | Dia155 | Head | 39 | 289 | 2485 | 9 | 8 | 114 | 16 |
| Dia003 | Dia006 | Head | 1 | 184 | 351 | 1 | 0 | 19 | 0 |
| Dia006 | Dia024 | Head | 6 | 627 | 1308 | 5 | 0 | 142 | 0 |
| Dia024 | Dia114 | Head | 0 | 281 | 319 | 0 | 0 | 20 | 0 |
| Dia114 | Dia144 | Head | 0 | 96 | 0 | 0 | 0 | 0 | 0 |
| Dia144 | Dia155 | Head | 104 | 1031 | 4753 | 25 | 23 | 496 | 39 |
| Dir000 | Dia000 | Abdomen | 0 | 85 | 350 | 0 | 0 | 12 | 0 |
| Dir000 | Dir003 | Abdomen | 7 | 166 | 875 | 0 | 0 | 27 | 4 |
| Dir000 | Dir006 | Abdomen | 53 | 292 | 1917 | 10 | 5 | 74 | 6 |
| Dir003 | Dir006 | Abdomen | 102 | 336 | 1236 | 17 | 10 | 54 | 10 |
| Dia000 | Dia003 | Abdomen | 0 | 103 | 468 | 0 | 0 | 14 | 0 |
| Dia000 | Dia006 | Abdomen | 2 | 150 | 1052 | 1 | 0 | 38 | 0 |
| Dia000 | Dia024 | Abdomen | 13 | 366 | 2480 | 1 | 5 | 141 | 5 |
| Dia000 | Dia114 | Abdomen | 12 | 516 | 2348 | 4 | 1 | 167 | 7 |
| Dia000 | Dia144 | Abdomen | 15 | 505 | 2536 | 3 | 4 | 180 | 6 |
| Dia000 | Dia155 | Abdomen | 20 | 326 | 2885 | 0 | 3 | 124 | 10 |
| Dia003 | Dia006 | Abdomen | 0 | 124 | 3 | 0 | 0 | 0 | 0 |
| Dia006 | Dia024 | Abdomen | 4 | 473 | 1047 | 4 | 0 | 83 | 0 |
| Dia024 | Dia114 | Abdomen | 0 | 247 | 233 | 0 | 0 | 16 | 0 |
| Dia114 | Dia144 | Abdomen | 1 | 116 | 0 | 0 | 0 | 0 | 0 |
| Dia144 | Dia155 | Abdomen | 29 | 741 | 3032 | 8 | 6 | 264 | 8 |

Table S6. **Genes in fuzzy c-means clusters of differentially expressed exons, events, and whole genes** (see attached spreadsheet; dimensions: 4 x 20037).

Table S7. **Gene set enrichment analysis of biological process GO terms performed by TopGO** for each of the fuzzy clusters of exon, event and whole gene expression. Significantly enriched terms were identified with two-way Fisher’s exact tests (parent-child algorithm) using a significance threshold of 0.01. (see attached spreadsheet; dimensions: 9 x 5149).

Table S8. **Gene set enrichment analysis of molecular function GO terms performed by TopGO** for each of the fuzzy clusters of exon, event and whole gene expression. Significantly enriched terms were identified with two-way Fisher’s exact tests (parent-child algorithm) using a significance threshold of 0.01. (see attached spreadsheet; dimensions: 9 x 685).

Table S9. **Kruskal-Wallis tests and effect sizes (η^2^) comparing differences among genes that were differentially spliced or expressed** in diapausing timepoints only (Diap.), both diapausing and direct time points (Both), among direct timepoints only (Dir.) and in genes without transcriptional variation (None). Tests were performed on full data and on 1000 matched samples (see attached spreadsheet; dimensions 14 x 72).

Table S10. **Dunn’s tests comparing differences between genes that were differentially spliced or expressed** in diapausing timepoints only (Diap.), both diapausing and direct time points (Both), among direct timepoints only (Dir.) and in genes without transcriptional variation (None). Tests were performed on full data and on 1000 matched samples (see attached spreadsheet; dimensions 13 x 432).

# Supplementary Figures


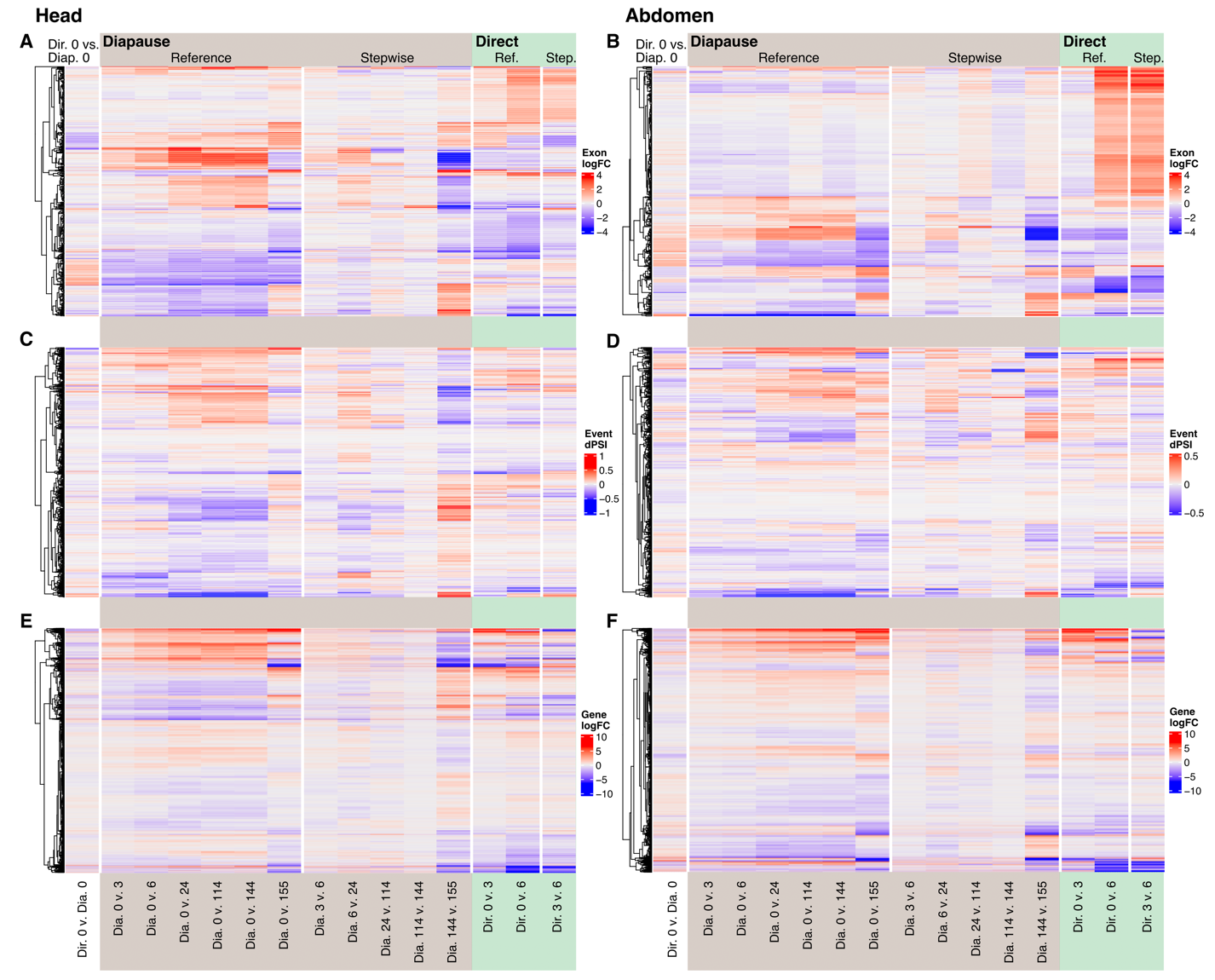


Fig. S1. **Degree of expression change of exons, events and whole genes** that were differentially spliced or expressed in the head and abdomen. Heatmaps show fold change (log_2_ transformed) in differentially expressed (A,B) exons and (E,F) whole genes, and difference in percent-spliced-in (dPSI) of (C, D) differentially expressed events. Expression was compared between diapause and direct developing pupae on day 0. Within developmental trajectories, expression was compared between each timepoint and day 0 (Reference analysis) and between adjacent timepoints (Stepwise analysis).

Fig. S2. **Standardized expression of exons** (divergence from the within-gene average of CPM-normalized exon expression, see methods) in genes identified as differentially spliced in the (A) head, and (B) abdomen, respectively. Total exons in each numbered cluster are indicated in parentheses. Within cluster, capped bars show 95% confidence intervals of event expression for each individual. Gene set enrichment analysis with topGO identified significantly enriched biological process (BP) and molecular function (MF) gene ontology terms (Table S3).

Fig. S3. **Standardized expression of splice events** (event inclusion level) in genes containing differentially expressed events in the (A) head, and (B) abdomen, respectively. Total events in each numbered cluster are indicated in parentheses. Within cluster, capped bars show 95% confidence intervals of event expression for each individual. Gene set enrichment analysis with topGO identified significantly enriched biological process (BP) and molecular function (MF) gene ontology terms (Table S3).

Fig. S4. **Standardized expression of whole genes (**CPM-normalized) in genes identified as differentially expressed in the (A) head, and (B) abdomen, respectively. Total genes in each numbered cluster are indicated in parentheses. Within cluster, capped bars show 95% confidence intervals of event expression for each individual. Gene set enrichment analysis with topGO identified significantly enriched biological process (BP) and molecular function (MF) gene ontology terms (Table S3)


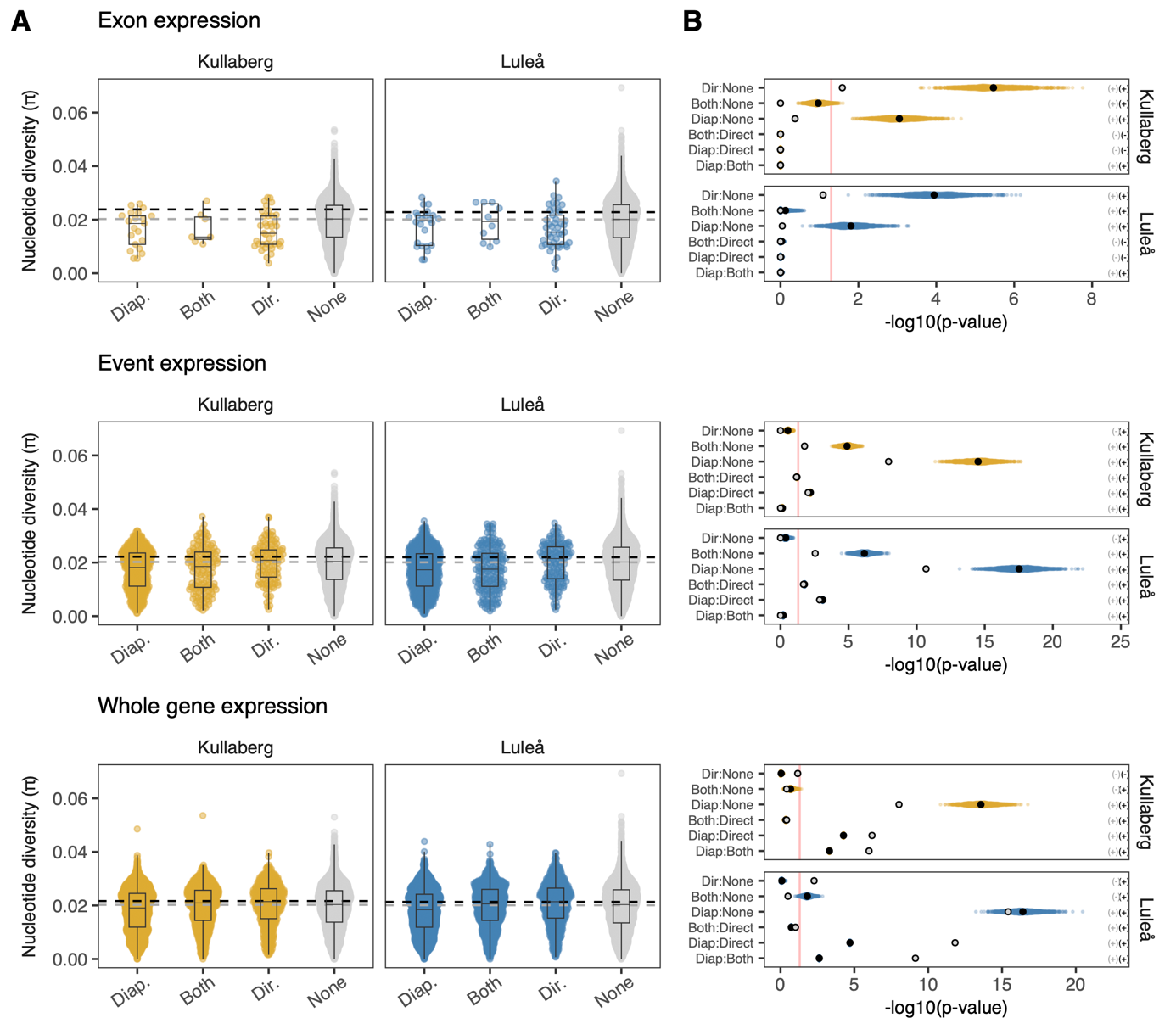


Fig. S5. **Nucleotide diversity (π) of genes with differential exon expression, event expression, and gene expression in the abdomen.** (A) π was compared among genes that were differentially spliced (DS) or expressed (DE) in diapausing timepoints only (Diap.), both diapausing and direct time points (Both), among direct timepoints only (Dir.) and in genes without transcriptional variation (None). Horizontal dashed lines represent median π for all ‘None’ genes (grey) and for matched gene sets (black). Matching the distribution of gene lengths, location along the chromosome (recombination rate proxy), and sample size with DS sets consistently increased the median nucleotide diversity in genes that were not DS (None). (B) Groups were compared with Kruskal-Wallis tests (Table S9) with Dunn’s post-hoc pairwise comparisons corrected for multiple tests within each set (6 tests; Table S10). Grey points show p-values (-log_10_ transformed) of Dunn’s multiple comparisons among full data, while yellow and blue points show p-values when comparing matched gene sets (n = 1000) for each population. Black points summarize means of these comparisons. The direction of the effect is shown in parentheses (grey = full data, black = matched ranges). Red vertical lines indicate a significance threshold of 0.05.


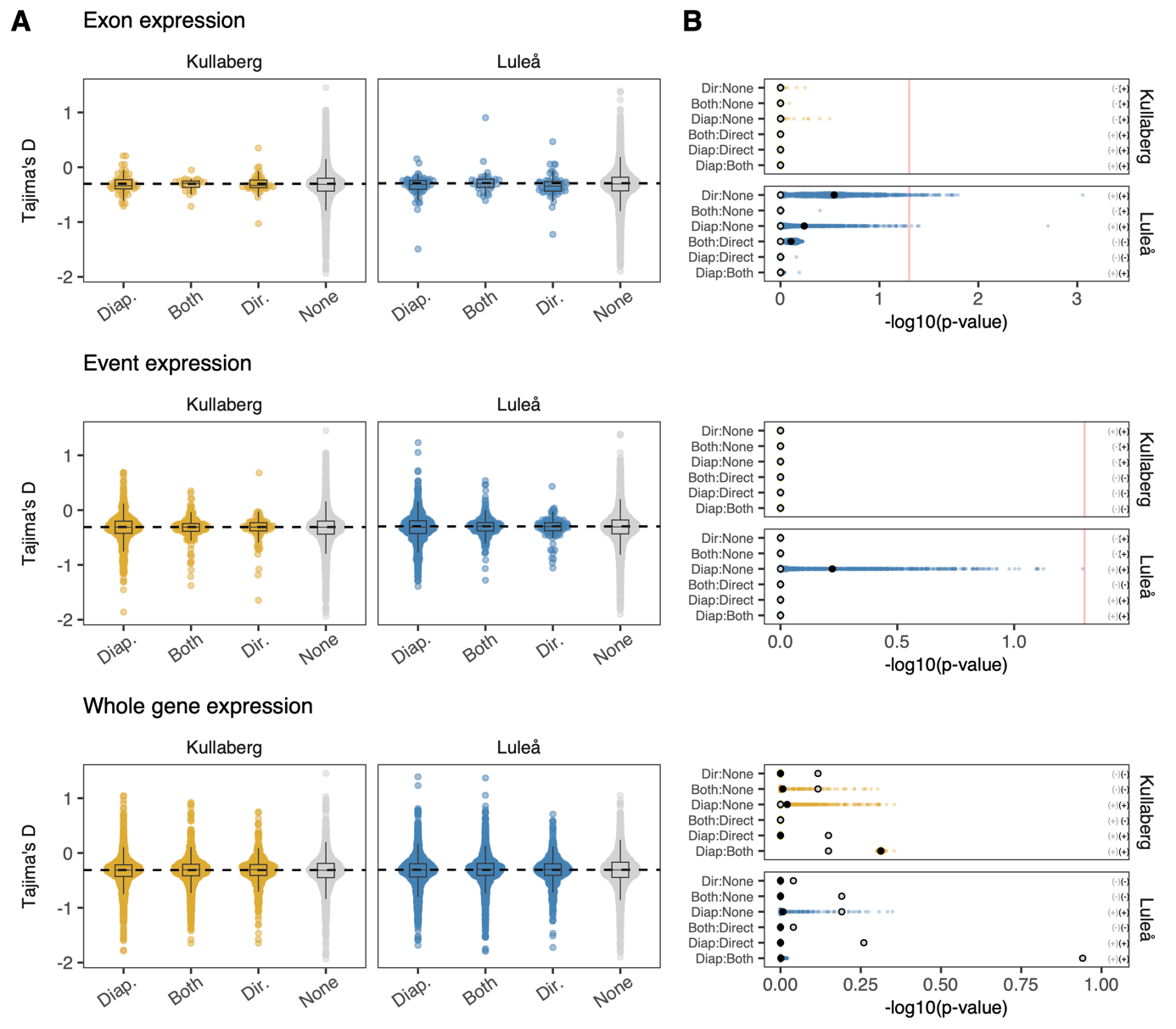


Fig. S6. **Tajima’s D of genes with differential exon expression, event expression, and gene expression in the head.** (A) Tajima’s D was compared among genes that were differentially spliced or expressed in diapausing timepoints only (Diap.), both diapausing and direct time points (Both), among direct timepoints only (Dir.) and in genes without transcription variation (None). In all cases, median Tajima’s D for all ‘None’ genes was equal to that of matched gene sets (black dashed line). (B) Groups were compared with Kruskal-Wallis tests (Table S9) with Dunn’s post-hoc pairwise comparisons corrected for multiple tests within each set (6 tests; Table S10). Grey points show p-values (-log_10_ transformed) of Dunn’s multiple comparisons among full data, while yellow and blue points show p-values when comparing matched gene sets (n = 1000) for each population. Black points summarize means of these comparisons. The direction of the effect is shown in parentheses (grey = full data, black = matched ranges). Red vertical lines indicate a significance threshold of 0.05.


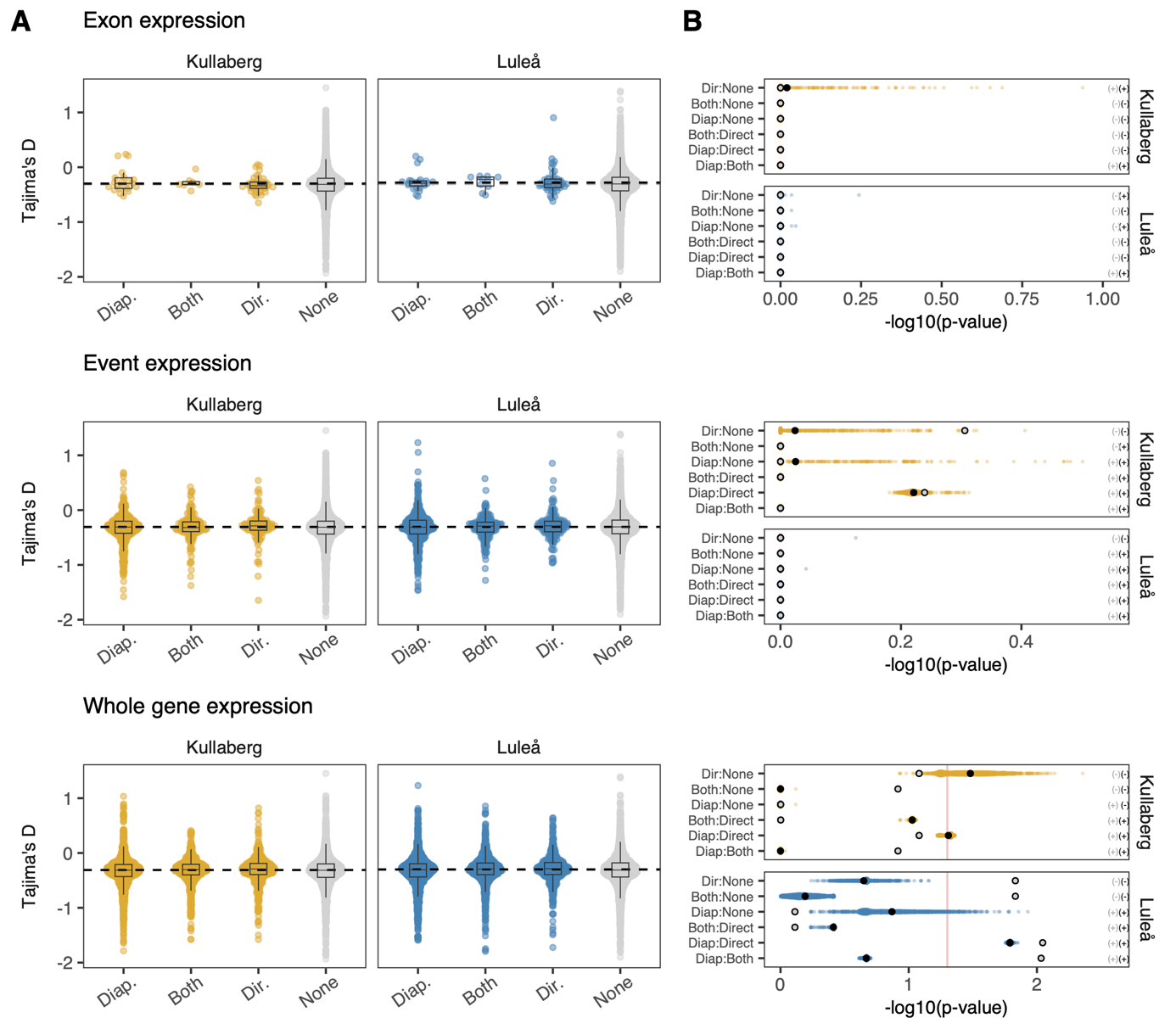


Fig. S7. **Tajima’s D of genes with differential exon expression, event expression, and gene expression in the abdomen.** (A) Tajima’s D was compared among genes that were differentially spliced or expressed in diapausing timepoints only (Diap.), both diapausing and direct time points (Both), among direct timepoints only (Dir.) and in genes without transcription variation (None). In all cases, median Tajima’s D for all ‘None’ genes was equal to that of matched gene sets (black dashed line). (B) Groups were compared with Kruskal-Wallis tests (Table S9) with Dunn’s post-hoc pairwise comparisons corrected for multiple tests within each set (6 tests; Table S10). Grey points show p-values (-log_10_ transformed) of Dunn’s multiple comparisons among full data, while yellow and blue points show p-values when comparing matched gene sets (n = 1000) for each population. Black points summarize means of these comparisons. The direction of the effect is shown in parentheses (grey = full data, black = matched ranges). Red vertical lines indicate a significance threshold of 0.05.


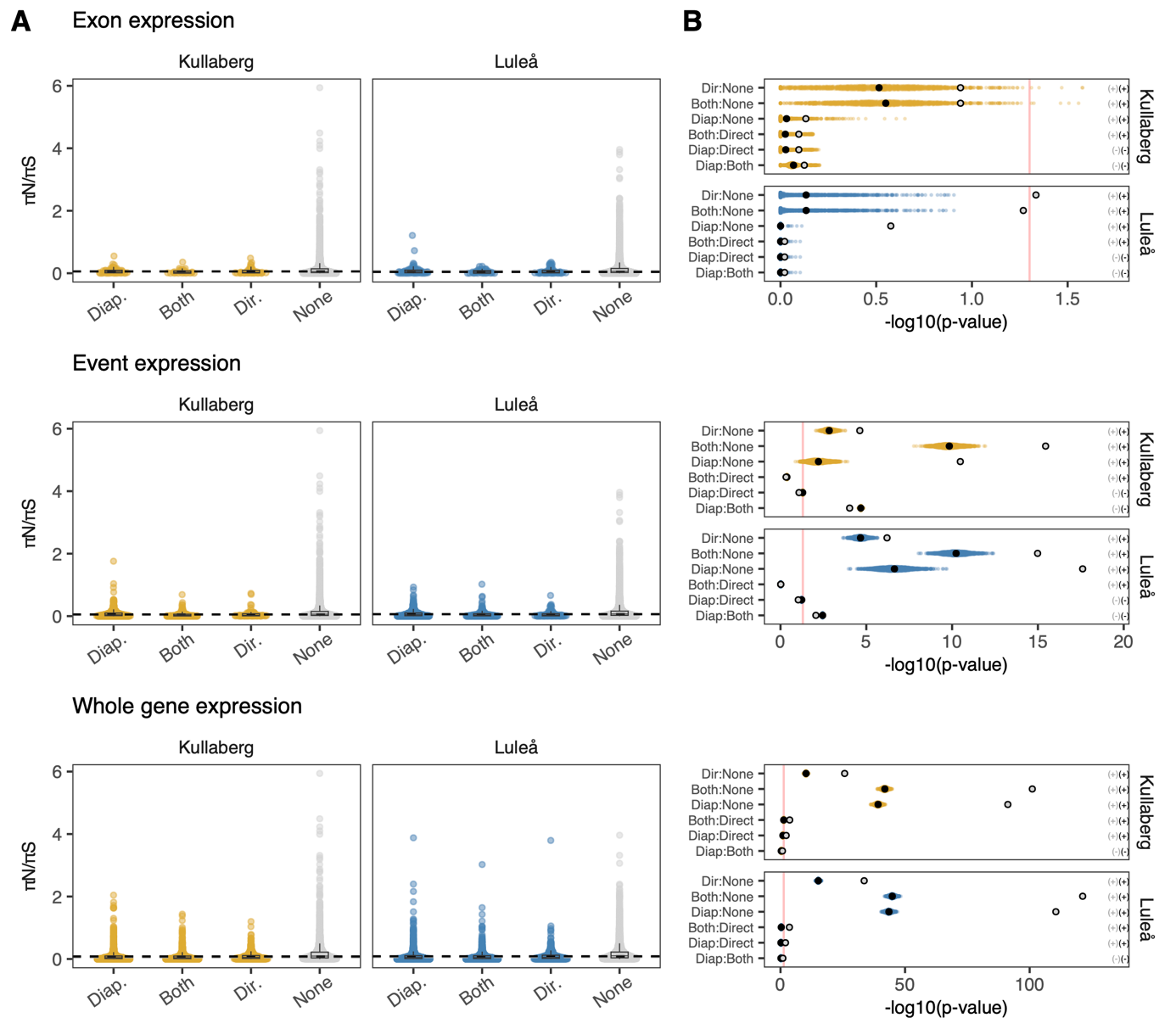


Fig. S8. **π_N_/π_S_ of genes with differential exon expression, event expression, and gene expression in the head.** (A) Tajima’s D was compared among genes that were differentially spliced or expressed in diapausing timepoints only (Diap.), both diapausing and direct time points (Both), among direct timepoints only (Dir.) and in genes without transcription variation (None). Median Tajima’s D for matched gene sets is shown with a black dashed line. (B) Groups were compared with Kruskal-Wallis tests (Table S9) with Dunn’s post-hoc pairwise comparisons corrected for multiple tests within each set (6 tests; Table S10). Grey points show p-values (-log_10_ transformed) of Dunn’s multiple comparisons among full data, while yellow and blue points show p-values when comparing matched gene sets (n = 1000) for each population. Black points summarize means of these comparisons. The direction of the effect is shown in parentheses (grey = full data, black = matched ranges). Red vertical lines indicate a significance threshold of 0.05.


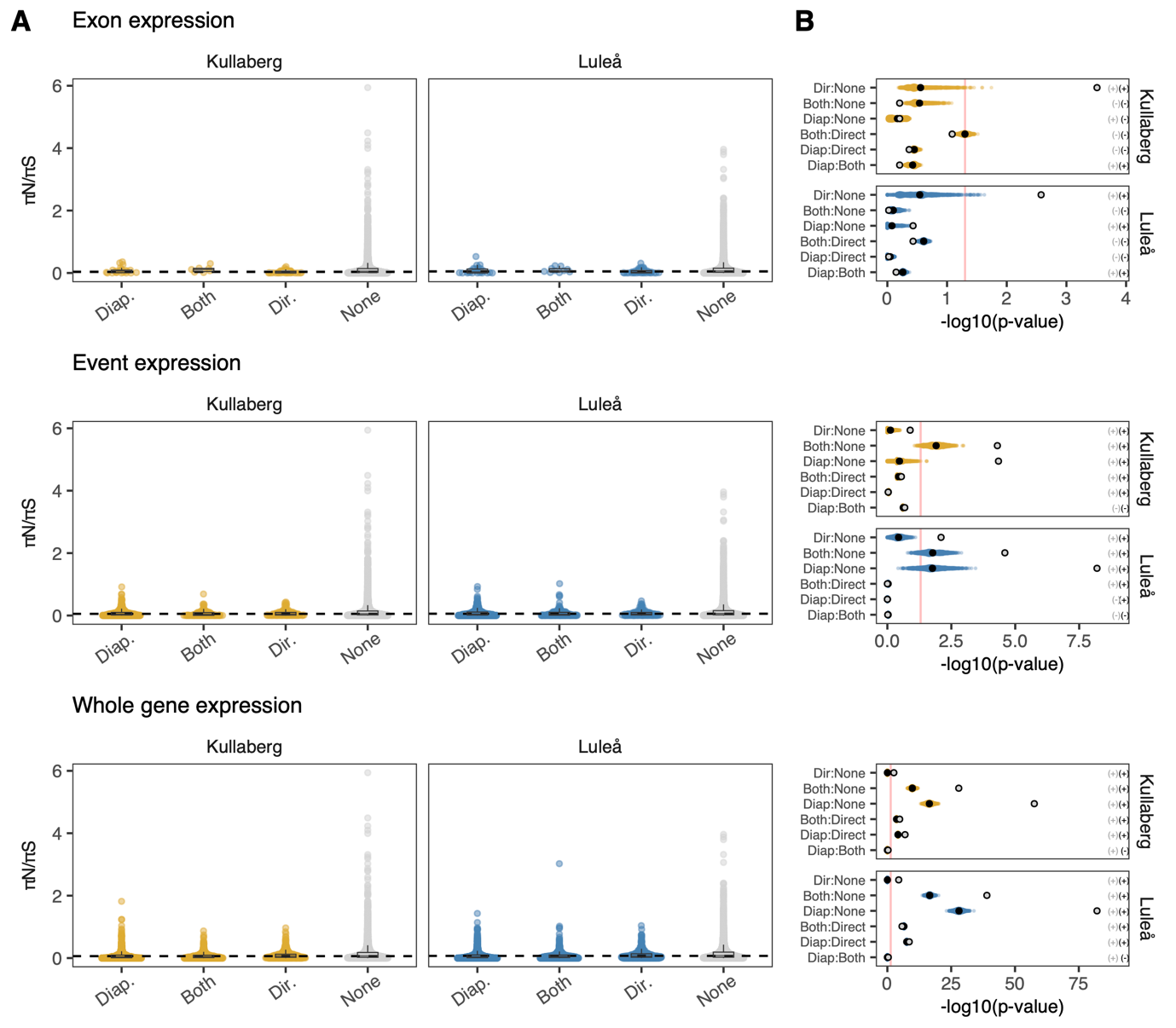


Fig. S9. **π_N_/π_S_ of genes with differential exon expression, event expression, and gene expression in the head.** (A) Tajima’s D was compared among genes that were differentially spliced or expressed in diapausing timepoints only (Diap.), both diapausing and direct time points (Both), among direct timepoints only (Dir.) and in genes without transcription variation (None). Median Tajima’s D for matched gene sets is shown with a black dashed line. (B) Groups were compared with Kruskal-Wallis tests (table SXXX) with Dunn’s post-hoc pairwise comparisons corrected for multiple tests within each set (6 tests). Grey points show p-values (-log_10_ transformed) of Dunn’s multiple comparisons among full data, while yellow and blue points show p-values when comparing matched gene sets (n = 1000) for each population. Black points summarize means of these comparisons. The direction of the effect is shown in parentheses (grey = full data, black = matched ranges). Red vertical lines indicate a significance threshold of 0.05.

# Supplementary References

Brůna T, Hoff KJ, Lomsadze A, Stanke M, Borodovsky M (2021). BRAKER2: Automatic Eukaryotic Genome Annotation with GeneMark-EP+ and AUGUSTUS Supported by a Protein Database. *NAR Genomics and Bioinformatics* **3**: lqaa108.

Buchfink B, Xie C, Huson DH (2015). Fast and sensitive protein alignment using DIAMOND. *Nat Methods* **12**: 59–60.

Dainat J, Hereñú D, Davis E, Crouch K, LucileSol, Agostinho N, *et al.* (2022). AGAT:Another GFF Analysis Toolkit to handle annotations in any GTF/GFF format.

Danecek P, Bonfield JK, Liddle J, Marshall J, Ohan V, Pollard MO, *et al.* (2021). Twelve years of SAMtools and BCFtools. *Gigascience* **10**: giab008.

Gabriel L, Hoff KJ, Brůna T, Borodovsky M, Stanke M (2021). TSEBRA: transcript selector for BRAKER. *BMC Bioinformatics* **22**: 566.

Hoff KJ, Lange S, Lomsadze A, Borodovsky M, Stanke M (2016). BRAKER1: Unsupervised RNA-Seq-Based Genome Annotation with GeneMark-ET and AUGUSTUS. *Bioinformatics* **32**: 767–769.

Hoff KJ, Lomsadze A, Borodovsky M, Stanke M (2019). Whole-Genome Annotation with BRAKER. In: Kollmar M (ed) *Gene Prediction: Methods and Protocols*, Methods in Molecular Biology. Springer: New York, NY, pp 65–95.

Kim D, Paggi JM, Park C, Bennett C, Salzberg SL (2019). Graph-based genome alignment and genotyping with HISAT2 and HISAT-genotype. *Nat Biotechnol* **37**: 907–915.

Li H, Handsaker B, Wysoker A, Fennell T, Ruan J, Homer N, *et al.* (2009). The Sequence Alignment/Map format and SAMtools. *Bioinformatics* **25**: 2078–2079.

Lohse K, Wright C, Talavera G, García-Berro A, Collective DT of LB, Programme WSIT of L, *et al.* (2021). The genome sequence of the painted lady, *Vanessa cardui* Linnaeus 1758.

Lomsadze A, Ter-Hovhannisyan V, Chernoff YO, Borodovsky M (2005). Gene identification in novel eukaryotic genomes by self-training algorithm. *Nucleic Acids Res* **33**: 6494–6506.

Manni M, Berkeley MR, Seppey M, Zdobnov EM (2021). BUSCO: Assessing Genomic Data Quality and Beyond. *Current Protocols* **1**: e323.

Stanke M, Diekhans M, Baertsch R, Haussler D (2008). Using native and syntenically mapped cDNA alignments to improve de novo gene finding. *Bioinformatics* **24**: 637–644.

Stanke M, Schöffmann O, Morgenstern B, Waack S (2006). Gene prediction in eukaryotes with a generalized hidden Markov model that uses hints from external sources. *BMC Bioinformatics* **7**: 62.

Ter-Hovhannisyan V, Lomsadze A, Chernoff YO, Borodovsky M (2008). Gene prediction in novel fungal genomes using an ab initio algorithm with unsupervised training. *Genome Res* **18**: 1979–1990.

Wright BW, Molloy MP, Jaschke PR (2022). Overlapping genes in natural and engineered genomes. *Nat Rev Genet* **23**: 154–168.
